# Maternal dietary quality, inflammatory potential and childhood adiposity: an individual participant data pooled analysis of seven European cohorts in the ALPHABET consortium

**DOI:** 10.1186/s12916-021-01908-7

**Published:** 2021-02-22

**Authors:** Ling-Wei Chen, Adrien M. Aubert, Nitin Shivappa, Jonathan Y. Bernard, Sara M. Mensink-Bout, Aisling A. Geraghty, John Mehegan, Matthew Suderman, Kinga Polanska, Wojciech Hanke, Agnieszka Jankowska, Caroline L. Relton, Sarah R. Crozier, Nicholas C. Harvey, Cyrus Cooper, Mark Hanson, Keith M. Godfrey, Romy Gaillard, Liesbeth Duijts, Barbara Heude, James R. Hébert, Fionnuala M. McAuliffe, Cecily C. Kelleher, Catherine M. Phillips

**Affiliations:** 1 HRB Centre for Health and Diet Research, School of Public Health, Physiotherapy, and Sports Science, University College Dublin, Dublin, Republic of Ireland; 2 Université de Paris, Centre for Research in Epidemiology and StatisticS (CRESS), Inserm, Inrae, F-75004 Paris, France; 3 Arnold School of Public Health, University of South Carolina, Columbia, SC 29208 USA; 4 Connecting Health Innovations, LLC, Columbia, SC 29201 USA; 5 Singapore Institute for Clinical Sciences (SICS), Agency for Science, Technology and Research (A*STAR), Singapore, 117609 Singapore; 6 The Generation R Study Group, Erasmus MC, University Medical Center Rotterdam, Rotterdam, The Netherlands; 7 Department of Pediatrics, Division of Respiratory Medicine and Allergology, Erasmus MC, University Medical Center Rotterdam, Rotterdam, The Netherlands; 8 UCD Perinatal Research Centre, School of Medicine, University College Dublin, National Maternity Hospital, Dublin, Ireland; 9 MRC Integrative Epidemiology Unit, Bristol Medical School, University of Bristol, Bristol, UK; 10 Nofer Institute of Occupational Medicine, Lodz, Poland; 11 MRC Lifecourse Epidemiology Unit (University of Southampton) University Hospital Southampton, Southampton, UK; 12 NIHR Southampton Biomedical Research Centre, University of Southampton and University Hospital Southampton NHS Foundation Trust, Southampton, UK; 13 Institute of Developmental Sciences, Faculty of Medicine, University of Southampton, Southampton, UK; 14 Department of Pediatrics, Erasmus MC, University Medical Center Rotterdam, Rotterdam, The Netherlands; 15 Department of Pediatrics, Division of Neonatology, Erasmus MC, University Medical Center Rotterdam, Rotterdam, The Netherlands

**Keywords:** Childhood obesity, Maternal, Pregnancy, Diet, Quality, Inflammation, Dietary inflammatory index, Dietary approaches to stop hypertension, Developmental origin of health and diseases

## Abstract

**Background:**

Mounting evidence suggests that maternal diet influences pregnancy and birth outcomes, but its contribution to the global epidemic of childhood obesity has not as yet been definitively characterized. We investigated whether maternal whole diet quality and inflammatory potential influence childhood adiposity.

**Methods:**

We harmonized and pooled individual participant data from 16,295 mother-child pairs in seven European birth cohorts. Maternal pre-, early-, late-, and whole-pregnancy (any time during pregnancy) dietary quality and inflammatory potential assessed with the Dietary Approaches to Stop Hypertension (DASH) score and the energy-adjusted Dietary Inflammatory Index (E-DII™) score, respectively. Primary outcome was childhood overweight and obesity (OWOB) (age-and-sex-specific BMI *z*-score > 85th percentile). Secondary outcomes were sum of skinfold thickness (SST), fat mass index (FMI) and fat-free mass index (FFMI). We used multivariable regression analyses (adjusting for maternal lifestyle and sociodemographic factors) to assess the associations of maternal DASH and E-DII scores with offspring adiposity outcomes in cohort-specific analyses, with subsequent random-effect meta-analyses.

**Results:**

The study mothers had a mean (SD) age of 30.2 (4.6) years and a mean BMI of 23.4 (4.2) kg/m^2^. Higher early-pregnancy E-DII scores (more pro-inflammatory diet) tended to be associated with a higher odds of late-childhood [10.6 (1.2) years] OWOB [OR (95% CI) 1.09 (1.00, 1.19) per 1-SD E-DII score increase], whereas an inverse association was observed for late-pregnancy E-DII score and early-childhood [2.8 (0.3) years] OWOB [0.91 (0.83, 1.00)]. Higher maternal whole pregnancy DASH score (higher dietary quality) was associated with a lower odds of late-childhood OWOB [OR (95% CI) 0.92 (0.87, 0.98) per 1-SD DASH score increase]; associations were of similar magnitude for early and late-pregnancy [0.86 (0.72, 1.04) and 0.91 (0.85, 0.98), respectively]. These associations were robust in several sensitivity analyses and further adjustment for birth weight and childhood diet did not meaningfully alter the associations and conclusions. In two cohorts with available data, a higher whole pregnancy E-DII and lower DASH scores were associated with a lower late-childhood FFMI in males and a higher mid-childhood FMI in females (*P* interactions < 0.10).

**Conclusions:**

A pro-inflammatory, low-quality maternal antenatal diet may adversely influence offspring body composition and OWOB risk, especially during late-childhood. Promoting an overall healthy and anti-inflammatory maternal dietary pattern may contribute to the prevention of childhood obesity, a complex health issue requiring multifaceted strategy.

**Supplementary Information:**

The online version contains supplementary material available at 10.1186/s12916-021-01908-7.

## Background

Childhood obesity has reached epidemic proportions worldwide [1]. Obesity in childhood has a pronounced impact on subsequent health risks as it often tracks into adulthood and is associated with a higher risk of chronic diseases including type 2 diabetes [2]. Although immediate lifestyle intervention in the paediatric population is essential, mounting evidence has pointed to the first 1000 days of life (from conception to 2 years old) as a critical period for preventing childhood obesity [3, 4]. In the 2016 Commission on Ending Childhood Obesity report commissioned by the World Health Organization (WHO), appropriate prenatal care such as optimizing maternal nutrition was highlighted as a key strategy to prevent childhood obesity [5].

The Developmental Origins of Health and Diseases (DOHaD) concept posits that early life represents a window of opportunity to optimize health trajectory of the immediate offspring and subsequent generations. Indeed, exposure to severe (in response to famine) or mild (e.g. suboptimal macronutrient composition) in utero malnutrition has been associated with a higher risk of obesity/higher adiposity [6–10] and metabolic disorders such as type 2 diabetes [11] later in life. Furthermore, maternal over-nutrition has also been shown to have a long-term influence on the offspring, including a higher risk of childhood obesity and other metabolic disorders (recently reviewed in [12]).

Due to the complexity of human diet [13] and because individuals do not consume nutrients and foods in isolation, the effectiveness of public health messaging to influence eating behaviours could be improved by assessing diet in a more holistic way. One such approach is through the use of diet quality indices based on previous scientific evidence that summarizes several aspects of dietary intake either against dietary guidelines or in terms of biological mechanism [14]. Because maternal inflammatory markers are associated with higher offspring adiposity [15, 16], reducing maternal inflammation through dietary optimization [17, 18] may be a potential strategy to reduce childhood obesity risk. A dietary inflammatory index (DII) has been designed specifically to measure diet-related inflammatory potential. DII likely captures a slightly different and narrower dimension of diet than a general dietary quality index, as suggested by only a moderate correlation between these indices [19]. Although maternal inflammation can be a mechanism through which maternal diet affects child health, other pathways such as epigenetic programming can also be responsible. Thus, by investigating maternal dietary inflammatory potential, we can infer the potential involvement of inflammation in our studied outcomes and make more specific dietary recommendations based on the DII if it does.

Most studies to date examining dietary quality or inflammatory potential and childhood adiposity have been conducted in a single country [20–23], potentially limiting generalizability because dietary habits could differ substantially across countries. These studies, in general, observed beneficial influence of higher maternal dietary quality and lower dietary inflammatory potential on offspring adiposity measures; however, one study reported null associations after adjusting for covariates [22]. We thus aimed to further elucidate the associations between maternal dietary quality, inflammatory potential and offspring adiposity outcomes through an individual participant data meta-analysis involving five countries.

A UK study that followed women from pre-conception through pregnancy showed that maternal dietary intakes and patterns changed little from before pregnancy to early and late pregnancy [24]. However, maternal diet at different stages of pregnancy could potentially differentially influence child outcomes, as such stages represent different phases of foetal growth and development. For example, foetal fat accretion starts only in late gestation [25]. We thus hypothesized that a pro-inflammatory and low-quality maternal diet is associated with a higher risk of childhood obesity, potentially with differential influences of maternal diet at different stages of preconception/pregnancy. Pre-conception diet has been increasingly associated with child outcomes, suggesting that there may be opportunities for interventions that change dietary patterns before conception to influence long-term child health [26]. Thus, in cohorts with available data, we also investigated potential influence of pre-pregnancy maternal dietary quality and inflammatory potential on childhood obesity.

## Methods

### Study population

This study involves seven mother-offspring cohort studies from five European countries within the ALPHABET consortium. These cohorts and longitudinal follow-up from a randomized controlled trial include the Lifeways Cross-Generation Cohort Study (Lifeways) (ISRCTN16537904) and the Randomised cOntrol trial of LOw glycaemic index diet during pregnancy study (ROLO) in Ireland (ISRCTN54392969); the study on the pre- and early postnatal determinants of child health and development (EDEN) in France; the Avon Longitudinal Study of Parents and Children (ALSPAC) and the Southampton Women’s Survey (SWS) in the UK; the Polish Mother and Child Cohort (REPRO_PL) (NCT01861548) in Poland; and The Generation R Study (Generation R) (NTR6671) in the Netherlands [27–35]. All studies have been approved by the respective local ethical review committees (see the ‘Acknowledgements’ section for details) and written consent was obtained from all mothers. The characteristics of each study and numbers of participants included in the current analysis are summarized in Additional file 1: Table S1 [27–35]. For more details of the respective cohorts, please refer to Additional file 1: Table S2 for a summary [36].

### Exposure

#### Maternal dietary assessment

Pre-pregnancy or antenatal dietary intakes of the study mothers were assessed using validated (except for ALSPAC which used a FFQ that had not been formally validated but covered the main foods consumed in Britain) food frequency questionnaires (FFQ) (mean food items in ALPHABET: 137), which have been described in detail elsewhere [37–45]. In ALPHABET, pre-pregnancy maternal diet was available in two studies (SWS and EDEN), while pregnancy maternal diet was assessed in all studies. Pregnancy diet was further classified based on a period of assessment: early pregnancy (1st/early 2nd trimester, *n* = 5 cohorts) and late pregnancy (3rd trimester, *n* = 3 cohorts). Since maternal diet was assessed during both early and late pregnancy in SWS, both were included and the average was taken to reflect whole pregnancy exposure. Whole pregnancy refers to dietary information assessed at any time point of pregnancy.

#### Derivation of maternal dietary inflammatory potential score

Maternal dietary inflammatory potential was ranked using the energy-adjusted Dietary Inflammatory Index (E-DII™), a well-validated literature-derived score described in detail elsewhere [46]. Briefly, dietary information for each mother was converted to amount per 1000-kcal intake and then linked to a regionally representative database. The regionally representative world composite database was constructed using national nutrition survey information across diverse populations living in a variety of countries in different regions of the world (USA, Australia, Bahrain, Denmark, India, Japan, New Zealand, Taiwan, South Korea, UK). The composite database provides a basis for benchmarking individual dietary intake to a representative range of dietary intake based on actual human consumption (more details available at [46]). The database provides an overall estimate of mean and standard deviation of energy-standardized intakes (using density method) for each of the dietary parameters (i.e. nutrients, foods, and other food components). Subsequently, *z*-scores for each dietary parameter were derived by subtracting the mean of the energy-adjusted regionally representative world composite database from the participants-reported amount and dividing this value by the parameter’s representative standard deviation. The *z*-scores were then converted to proportions (i.e. with values ranging from 0 to 1) and then centred by doubling and subtracting 1. The resulting value was then multiplied by the corresponding food parameter-specific inflammatory effect score (derived from a comprehensive literature review of 1943 peer-reviewed articles) and summed to yield the overall E-DII score. A higher E-DII score indicates a more pro-inflammatory diet. The systematic review was conducted through a comprehensive search of the National Library of Medicine database from 1950 through 2010, and the literature search strategy along with inclusion criteria were described in detail in the DII development paper [46]. The maternal E-DII score in ALPHABET was generated from 24 to 28 dietary parameters (out of 44 possible; as energy intake has been intrinsically adjusted for in E-DII, it is not considered as a separate parameter) in all cohorts except for Generation R, which has 20 dietary parameters (see Additional file 1: Table S3). In available cohorts (except EDEN), child E-DII scores were also generated. When calculated from all the 45 possible food parameters (including energy intake), DII scores ranged from − 8.87 (most anti-inflammatory) to + 7.98 (most pro-inflammatory); however, when derived from 25 to 30 food parameters, the range seen for many population is from − 5.5 to + 5.5 [19]. In our constituent studies, the range of E-DII is within − 5.5 to + 5.5, supporting that they have been calculated correctly. The range for Generation R using only 20 parameters appears comparable to other cohorts (see Additional file 1: Fig. S1). We thus believe that the E-DII score captures dietary inflammatory potential sufficiently in our study.

#### Derivation of maternal dietary quality score

Dietary quality was assessed by degree of adherence to the Dietary Approaches to Stop Hypertension (DASH) diet. The moderation and harmonization process for DASH score generation within the ALPHABET consortium has been described elsewhere [36]. The derivation of the DASH score in ALPHABET was based mainly on the index proposed by Fung et al. [47], which ranks an individual’s diet based on population quintile ranking. We deemed Fung et al.’s approach more suitable for our FFQ derived data, which aims to rank participants according to their intakes rather than absolute estimation of food intakes, as compared with other ranking approaches based on whether one meets recommended servings of foods [48]. The final ALPHABET DASH score comprised 8 components (see Additional file 1: Table S4). For food components with higher intake recommended (fruits, vegetables excluding potatoes, total grains, non-full-fat dairy products, and nuts/seeds/legumes), participants in the highest quintile received a score of 5 while those in the lowest quintile received a score of 1. Reverse scoring was applied to food components with moderation recommended (red and processed meats, sugar-sweetened beverages/sweets/added sugars, and sodium). The DASH score has a theoretical range of 8 to 40, with a higher score reflecting a higher dietary quality.

### Outcomes

The primary outcome was childhood overweight and obesity (OWOB), defined as age- and sex-specific body mass index *z* score (BMI *z*) > 85th percentile [49]. BMI was calculated from reported or measured weight and height using the formula weight (in kg)/height (in m^2^), which was subsequently converted to *z*-scores using the WHO Child Growth Standards for 0–5 years [50] and 5–19 years [51]. Secondary outcomes were sum of skinfold thickness (SST; subscapular skinfold + triceps skinfold, mm), fat mass index (FMI, kg/m^2^) and fat-free mass index (FFMI, kg/m^2^) in available cohorts (see Additional file 1: Table S5 for outcome data availability). Body composition measures used in this study were based on bioelectrical impedance analysis, except for SWS, which used dual-energy X-ray absorptiometry. Outcomes were assessed in early (pre-school) [mean (SD) age: 2.8 (0.3) years], mid [6.1 (0.6) years] and late-childhood [10.6 (1.2) years] [52, 53].

### Covariates

Potential confounders and relevant covariates were identified from literature and harmonized for subsequent analysis. These were maternal age at delivery (in years), maternal height (in cm), pre-pregnancy BMI (in kg/m^2^), maternal educational status (study-specific definition of low/medium/high), self-reported maternal birthplace/ethnicity (European-born/White or non-European-born/non-White), maternal cigarette smoking [never/ever (stopped during the pregnancy)/current (continued during the pregnancy)], maternal alcohol intake during pregnancy (yes/no), maternal parity (primiparous/multiparous) and child exact age at anthropometry measurement (in months) and sex (male/female). These data were originally abstracted from birth records or collected using questionnaires (interviewer- or self-administered).

### Statistical analysis

Participants’ characteristics were summarized for the ALPHABET consortium and according to its constituent studies. These were limited to participants with availability of the exposure (maternal diet) and main outcome (childhood BMI measurement at any time point) variables. We further excluded participants (*n* = 396) with likely implausible energy intakes (< 500 or > 3500 kcal/day) to avoid extreme misreporting [54, 55].

A two-stage individual participant data meta-analysis was used to assess the associations between maternal diet quality and inflammatory potential and childhood adiposity outcomes. First, cohort-specific effect estimates were generated by using linear and logistic regression for continuous and binary outcomes, respectively. The effect estimates were subsequently pooled using random-effects meta-analysis, which considers both within- and between-study variability. Cochran’s *Q* test and *I*^2^ statistic were used to assess statistical heterogeneity among included studies [56, 57].

The aforementioned a priori selected covariates were adjusted. Missing covariate information was imputed using cohort-specific means (continuous variables) or modal categories (categorical variables). Complete case analysis yielded largely similar results and did not affect study conclusions (results not shown).

We also conducted several sensitivity analyses. First, we limited our analysis to European-born/White participants (which make up 89% of the study population) to reduce heterogeneity in participants' characteristics. Second, we excluded participants with gestational diabetes, gestational hypertension and pre-eclampsia to see if associations persist in a subset of relatively low-risk pregnancies. Third, a stricter definition of obesity was used (> 95th percentile of age- and sex-specific *z*-score) to investigate whether maternal dietary quality and inflammatory potential can impact more severe childhood obesity. To explore potential mechanism related to inflammation, we mutually adjusted for DASH and E-DII scores in the same model. For statistically significant associations, we further tested potential mediation through dietary inflammation or dietary quality using mixed effects (with random intercept for each cohort) causal mediation model through the ‘Mediation’ package in R version 3.6.3 (R Foundation for Statistical Computing, Vienna, Austria). Because we previously showed that a low quality and pro-inflammatory maternal diet was associated with lower birth size [58], we further investigated whether birthweight is a potential mediator in the current study. As with birth weight, gestational age is also known to influence both growth and body composition in childhood, so we conducted analysis further adjusting for gestational age. Child E-DII scores were available in all cohorts except EDEN and were also examined as a potential mediator for maternal diet vs late-childhood adiposity relationships (because child E-DII scores were mostly examined during mid to late-childhood). Child E-DII score was used over child DASH score (not generated) due to logistical constraints, because childhood diet was not the main focus of the ALPHABET consortium. Most cohorts also used a shortened FFQ for child dietary intake assessment, causing an insufficient number of food items in several food groups for DASH score generation (DII is less affected because it has predominantly nutrient parameters). In addition, although the child DII score has been developed and validated [59], we are not aware of similar validation being conducted for a child version of the DASH score.

We investigated whether child sex was a potential modifier of the associations of maternal dietary quality and inflammatory potential with offspring adiposity outcomes by including the multiplicative interaction terms into the model one at a time. This was done using participant-level data for each cohort, and the estimates were subsequently pooled. When *P*-interaction was < 0.10, downstream stratification analyses were conducted.

All analyses, unless otherwise specified, were performed using the statistical software Stata version 13.1 (StataCorp, College Station, TX, USA), and statistical significance was defined as two-sided *P* values < 0.05. Because many statistical tests were conducted, the threshold of *P* < 0.05 is appropriate for analyses concerning our primary outcome i.e. childhood OWOB. Other analyses involving secondary outcomes, albeit reaching statistical significance, should be considered as hypothesis generating and such findings need to be replicated in further studies.

## Results

The current analysis included up to 16,295 mother-child pairs from seven European studies. Overall, the study mothers had a mean (SD) age of 30.2 (4.6) years at delivery and a mean BMI of 23.4 (4.2) kg/m^2^ (Additional file 1: Table S1). Mean (SD) [range] of dietary scores were: pre-pregnancy E-DII = 0.0 (1.7) [− 4.7 to 4.3]; pregnancy E-DII = 0.1 (1.6) [− 5.4 to 5.5]; pre-pregnancy DASH = 24.3 (4.3) [10 to 37]; and pregnancy DASH = 24.3 (4.2) [10 to 38]. Pearson’s correlation coefficients between E-DII and DASH were − 0.59 for pre-pregnancy and − 0.49 during pregnancy (both *P* < 0.001) (also see Additional file 1: Fig. S2 for scatter plots). The percentages of children classified as OWOB ranged from 8.1% (EDEN) to 21.6% (ALSPAC) during early-childhood, 6.4% (EDEN) to 23.8% (Lifeways) during mid-childhood and 7.0% (EDEN) to 19.1% (Lifeways) during late-childhood.

### Associations of E-DII and DASH scores with primary outcome (OWOB)

**Fig. 1:**
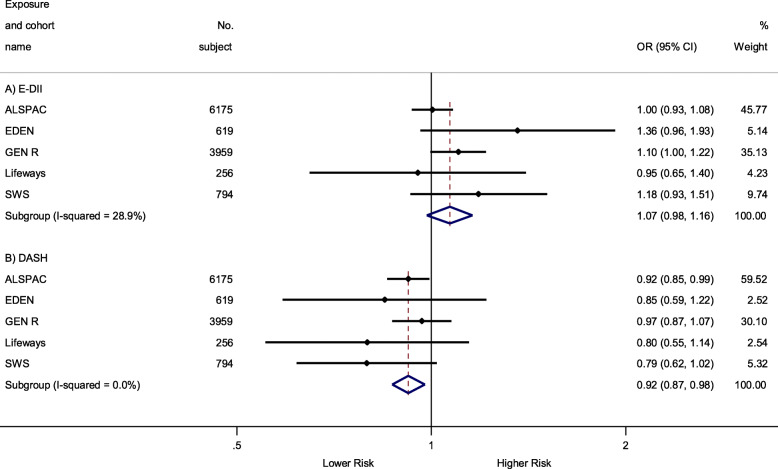
Associations between maternal pregnancy E-DII and DASH scores and late-childhood [10.6 (1.2) years] OWOB. Black dots indicate study-specific point effect estimates with corresponding 95% CIs indicated by horizontal lines, and diamonds indicate the pooled estimates with their corresponding 95% CIs. When studies were omitted one at a time for pregnancy E-DII and DASH meta-analysis, the overall pooled estimates were largely the same: for E-DII, pooled estimates ranged from 1.05 (0.98, 1.12) when excluding EDEN to 1.12 (1.02, 1.22) when excluding ALSPAC; for DASH, the pooled estimates ranged from 0.93 (0.88, 0.99) when excluding SWS to 0.90 (0.84, 0.97) when excluding GEN R. Effect estimates were adjusted for maternal education, ethnicity, pre-pregnancy BMI, maternal height, parity, energy intake (for DASH analysis only), cigarette smoking and alcohol consumption during pregnancy and (intrinsically adjusted for the outcome) child sex and age at measurement. E-DII, energy-adjusted Dietary Inflammatory Index; DASH, Dietary Approach to Stop Hypertension; OWOB, overweight and obesity

The main associations (involving greatest numbers of mother-child pairs) between maternal whole pregnancy E-DII and DASH scores and late-childhood OWOB are summarized in Fig. 1. A higher maternal DASH score (higher dietary quality) during whole pregnancy was associated with a lower odds of late-childhood OWOB [OR (95% CI) 0.92 (0.87, 0.98) per 1-SD increase in DASH score], whereas no statistically significant association was observed for whole-pregnancy E-DII score [OR (95% CI) 1.07 (0.98, 1.16) per 1-SD increase in E-DII score; *P* > 0.05]. These associations were of similar magnitude for early- and late-pregnancy (instead of whole-pregnancy) dietary scores with slight variations in statistical significance (Table 1).

**Table 1:** Associations between maternal E-DII and DASH scores (per 1-SD increase) and childhood OWOB (BMI *z*-score > 85th percentile)

	Early-childhood		Mid-childhood		Late-childhood	
	OR (95%CI)	*I*^2^ (%)	OR (95% CI)	*I*^2^ (%)	OR (95% CI)	*I*^2^ (%)
**E-DII**						
Pre	0.94 (0.84, 1.05)	0	1.01 (0.89, 1.15)	0	0.96 (0.81, 1.15)	0
Np/Nc	3122/2		2635/2		1658/2	
Preg	0.94 (0.87, 1.02)	0	0.97 (0.91, 1.03)	0	1.07 (0.98, 1.16)	29
Np/Nc	6111/6		8717/7		11,803/5	
Early	0.98 (0.89, 1.07)	0	0.97 (0.91, 1.04)	0	1.09 (0.999, 1.19)	0
Np/Nc	4103/4		6903/5		5063/3	
Late	0.91 (0.83, 0.997)*	0	0.97 (0.85, 1.11)	21	1.07 (0.93, 1.23)	41
Np/Nc	4027/3		3419/3		7779/3	
**DASH**						
Pre	0.98 (0.80, 1.22)	62	0.93 (0.81, 1.06)	0	0.96 (0.80, 1.16)	0
Np/Nc	3122/2		2635/2		1658/2	
Preg	0.98 (0.91, 1.06)	0	1.03 (0.96, 1.11)	3	0.92 (0.87, 0.98)**	0
Np/Nc	6111/6		8717/7		11,803/5	
Early	0.99 (0.90, 1.09)	0	1.05 (0.97, 1.14)	7	0.86 (0.72, 1.04)	55
Np/Nc	4103/4		6903/5		5065/3	
Late	1.01 (0.92, 1.11)	0	0.96 (0.85, 1.07)	0	0.91 (0.85, 0.98)*	0
Np/Nc	4027/3		3419/3		7779/3	

Values are adjusted pooled effect estimates [OR (95% CI)] expressed for a 1-SD increment in dietary scores, heterogeneity measure (*I*^2^) and the number of participants and studies included (Np/Nc) across different outcomes and conception periods, as labelled. Effect estimates were adjusted for maternal education, ethnicity, pre-pregnancy BMI, maternal height, parity, energy intake (for DASH analysis only), cigarette smoking and alcohol consumption during pregnancy and (intrinsically adjusted for the outcome) child sex and age at measurement

OR (effect estimates) and the *I*^2^ (heterogeneity) test have separate *P* values

*E-DII* energy-adjusted Dietary Inflammatory Index, *DASH* Dietary Approaches to Stop Hypertension, *OWOB* overweight and obesity, *I*^2^ *I*-squared, *Pre* pre-pregnancy, *Preg* pregnancy, *Early* early pregnancy, *Late* late pregnancy, *Np* number of participants included, *Nc* number of cohorts included

*P < 0.05, **P < 0.01

In general, no consistent associations were observed between maternal E-DII and DASH scores with regards to early- and mid-childhood OWOB. The only exception is that higher late-pregnancy E-DII score was associated with a lower odds of early-childhood OWOB [OR (95% CI) 0.91 (0.83, 1.00)].

### Associations of E-DII and DASH scores with secondary adiposity measures

Similar to the primary outcome, associations between maternal dietary scores and secondary adiposity measures were only observed during late-childhood (Table 2). A higher whole pregnancy E-DII score was associated with a lower late-childhood FFMI [*β* (95% CI − 0.06 (− 0.08, − 0.03) kg/m^2^ per 1-SD increase in E-DII score]. In contrast, a higher whole pregnancy DASH score was associated with a lower late-childhood FMI [*β* (95% CI − 0.09 (− 0.15, − 0.03) kg/m^2^]. Across different pregnancy periods, the point estimates were in the same direction and of comparable magnitude, though the sample sizes and statistical significance varied (some analyses include only 1 study). No other apparent associations were observed for other periods or between maternal E-DII, DASH scores and childhood SST.

**Table 2:** Associations between maternal E-DII and DASH scores (per 1-SD increase) and secondary childhood adiposity measures

	Early-childhood (2.8 ± 0.3 years)		Mid-childhood (6.1 ± 0.6 years)						Late-childhood (10.6 ± 1.2 years)					
	SST, mm		SST, mm		FMI, kg/m^2^		FFMI, kg/m^2^		SST, mm		FMI, kg/m^2^		FFMI, kg/m^2^	
	*β* (95% CI)	*I*^2^ (%)	*β* (95% CI)	*I*^2^ (%)	*β* (95% CI)	*I*^2^ (%)	*β* (95% CI)	*I*^2^ (%)	*β* (95% CI)	*I*^2^ (%)	*β* (95% CI)	*I*^2^ (%)	*β* (95% CI)	*I*^2^ (%)
**E-DII**														
Pre	− 0.11 (−0.23, 0.01)	0	− 0.04 (− 0.22, 0.14)	0	− 0.01 (− 0.04, 0.03)	0	− 0.04 (− 0.08, 0.003)	0	− 0.17 (− 0.76, 0.41)	–	0.03 (− 0.10, 0.16)	–	− 0.03 (−-0.10, 0.05)	–
Np/Nc	2959/2		2536/2		2034/2		2038/2		1025/1		848/1		848/1	
Preg	− 0.01 (− 0.14, 0.13)	13	0.09 (− 0.14, 0.33)	29	0.03 (− 0.03, 0.09)	25	− 0.06 (− 0.16, 0.03)	67*	0.41 (− 0.23, 1.06)	–	0.03 (− 0.03, 0.10)	0	− 0.06 (− 0.08, − 0.03)***	0
Np/Nc	2749/3		3184/4		2059/4		2063/4		780/1		6739/2		6739/2	
Early	0.07 (− 0.10, 0.24)	0	− 0.01 (− 0.28, 0.25)	0	0.01 (− 0.10, 0.13)	41	− 0.13 (− 0.32, 0.06)	77*	0.20 (− 0.43, 0.82)	–	0.04 (− 0.09, 0.17)	–	− 0.04 (− 0.12, 0.04)	–
Np/Nc	1716/2		1513/2		1120/3		1119/3		836/1		702/1		702/1	
Late	− 0.04 (− 0.17, 0.08)	0	0.15 (− 0.07, 0.38)	17	0.03 (− 0.01, 0.07)	0	− 0.001 (− 0.04, 0.04)	0	0.31 (− 0.28, 0.90)	–	0.04 (− 0.03, 0.11)	12	− 0.04 (− 0.10, 0.01)	52
Np/Nc	2880/2		3182/3		1980/2		1984/2		969/1		6885/2		6885/2	
**DASH**														
Pre	− 0.03 (− 0.16, 0.09)	2	− 0.18 (− 0.36, 0.01)	0	− 0.04 (− 0.08, 0.003)	0	0.03 (− 0.01, 0.08)	0	− 0.29 (− 0.88, 0.31)	–	− 0.10 (− 0.23, 0.03)	–	0.05 (− 0.03, 0.12)	–
Np/Nc	2959/2		2536/2		2034/2		2038/2		1025/1		848/1		848/1	
Preg	− 0.06 (− 0.18, 0.07)	0	− 0.02 (− 0.29, 0.26)	41	− 0.02 (− 0.05, 0.02)	0	0.01 (− 0.03, 0.06)	0	− 0.54 (− 1.21, 0.12)	–	− 0.09 (− 0.15, − 0.03)**	0	0.03 (− 0.002, 0.06)	0
Np/Nc	2749/3		3184/4		2059/4		2063/4		780/1		6739/2		6739/2	
Early	− 0.10 (− 0.28, 0.08)	0	0.10 (− 0.41, 0.61)	68	− 0.07 (− 0.15, 0.01)	0	0.03 (− 0.03, 0.09)	0	− 0.58 (− 1.22, 0.06)	–	− 0.12 (− 0.25, 0.01)	–	0.06 (− 0.02, 0.14)	–
Np/Nc	1716/2		1513/2		1120/3		1119/3		836/1		702/1		702/1	
Late	− 0.01 (− 0.14, 0.11)	0	− 0.06 (− 0.24, 0.13)	0	− 0.01 (− 0.05, 0.03)	0	− 0.001 (− 0.05, 0.04)	0	− 0.28 (− 0.88, 0.32)	–	− 0.07 (− 0.13, − 0.01)*	0	0.03 (− 0.002, 0.05)	0
Np/Nc	2880/2		3182/3		1980/2		1984/2		969/1		6885/2		6885/2	

Values are adjusted pooled effect estimates [*β* (95% CI)] expressed for a 1-SD increment in dietary scores, heterogeneity measure (*I*^2^), and number of participants and studies included (Np/Nc) across different outcomes and conception periods, as labelled. Effect estimates were adjusted for maternal education, ethnicity, pre-pregnancy BMI, maternal height, parity, energy intake (for DASH analysis only), cigarette smoking and alcohol consumption during pregnancy and child sex and age at measurement

*β* (effect estimates) and the *I*^2^ (heterogeneity) test have separate *P* values

*E-DII* energy-adjusted Dietary Inflammatory Index, *DASH* Dietary Approaches to Stop Hypertension, *SST* sum of skinfold thickness, *FMI* fat mass index, *FFMI* fat-free mass index, *I*^2^ *I*-squared, *Pre* pre-pregnancy, *Preg* pregnancy, *Early* early pregnancy, *Late* late pregnancy, *Np* number of participants included, *Nc* number of cohorts included

*P < 0.05, **P < 0.01, ***P < 0.001

### Sensitivity analyses

When non-European-born/non-White participants were excluded, the aforementioned associations were stronger (i.e. estimates moved away from null) (Additional file 1: Table S6 and Table S7). In contrast, there was a slight attenuation between maternal DASH and late-childhood OWOB (but not late-childhood FMI) when mothers with pregnancy complications were removed (Additional file 1: Table S8 and Table S9) or in models with mutual adjustment of the dietary scores (Additional file 1: Table S10 and Table S11); however, the overall patterns of associations remained the same. Further causal mediation analyses showed little evidence of mediation through the other dietary score for the significant associations between whole pregnancy dietary quality and inflammation and late childhood OWOB/adiposity. The proportions mediated by dietary inflammation on higher dietary quality vs. lower FMI relationship was 5.5% (*P* = 0.73) and that on higher dietary quality vs. lower OWOB odds was 13.1% (*P* = 0.48). For the significant association between more pro-inflammatory diet and lower FFMI, the proportion mediated by dietary quality was 7.0% (*P* = 0.69). When a stricter childhood obesity outcome was used (> 95th sex-and-age-specific BMI *z*-score), most of the abovementioned point estimates were stronger, except for late-pregnancy E-DII and early-childhood obesity which was largely attenuated (Additional file 1: Table S12); the confidence intervals became overall wider, probably due to the smaller number of cases. When birthweight was included in the model, the estimates changed little (Additional file 1: Table S13 and Table S14), suggesting little mediation by birth size. Similarly, results remained the same with adjustment for gestational age at birth (Additional file 1: Table S15 and Table S16). Associations also remained very similar when all children born small for gestational age were excluded (results not shown). In cohorts with child E-DII scores, further adjusting for child dietary inflammatory potential also led to very similar estimates (Additional file 1: Table S17), and the association reported for higher maternal E-DII vs. lower late-childhood FFMI remained statistically significant. Similarly, results remained essentially unchanged with further adjustment for whether the child was ever breastfed (results not shown).

### Sex-stratified analysis

**Fig. 2:**
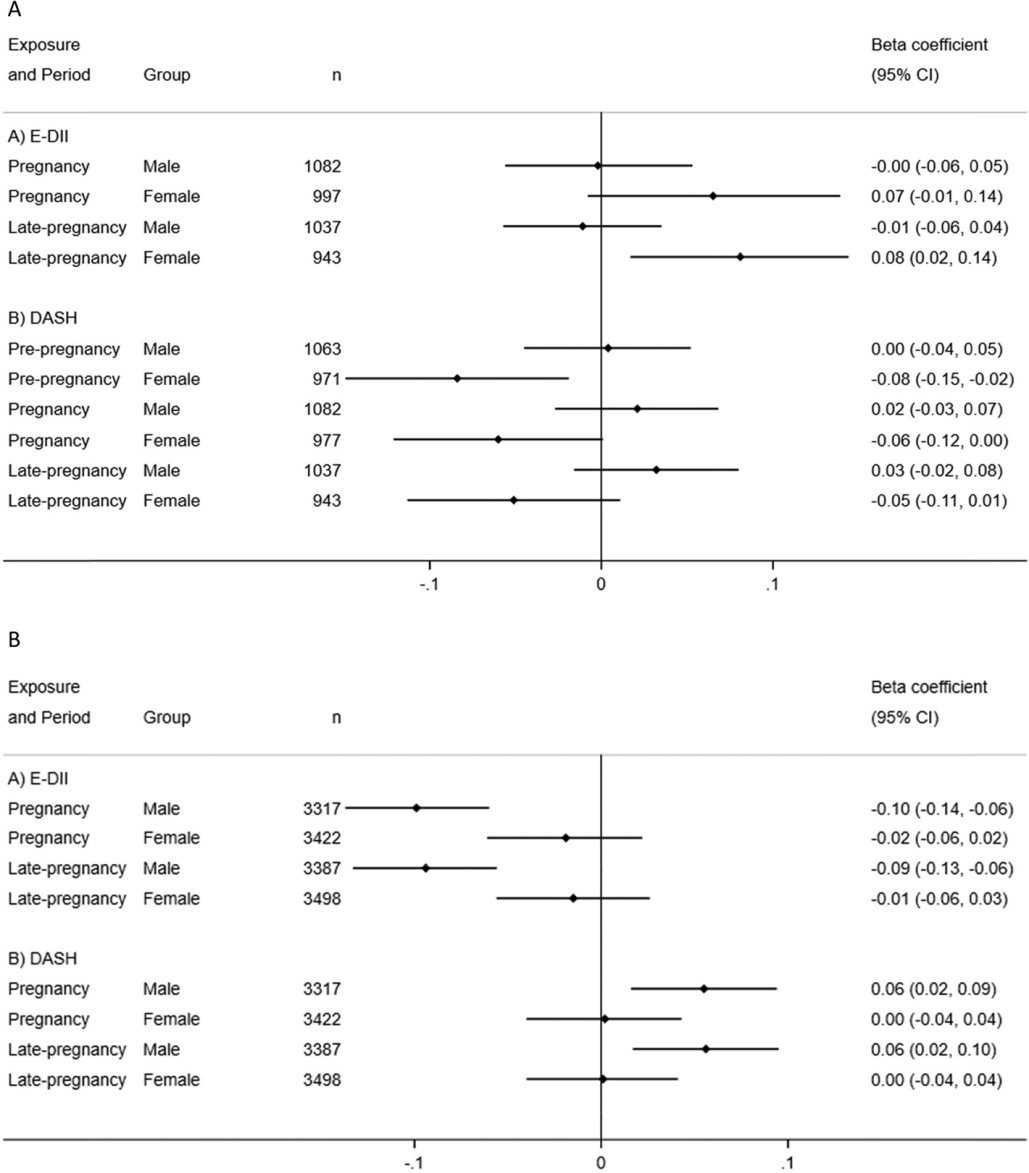
**a** Associations of maternal E-DII and DASH scores with children’s FMI during mid-childhood by child sex and exposure time-point (only adjusted pooled estimates shown; all *P*-interactions < 0.10). Black dots indicate pooled point effect estimates with corresponding 95% CIs indicated by horizontal lines. Effect estimates were adjusted for maternal education, ethnicity, pre-pregnancy BMI, maternal height, parity, energy intake (for DASH analysis only), cigarette smoking and alcohol consumption during pregnancy and child sex and age at measurement. E-DII, energy-adjusted Dietary Inflammatory Index; DASH, Dietary Approaches to Stop Hypertension; FMI, fat mass index. **b** Associations of maternal E-DII and DASH scores with children’s FFMI during late-childhood by child sex and exposure time-point (only adjusted pooled estimates shown; all *P*-interactions < 0.10). Black dots indicate pooled point effect estimates with corresponding 95% CIs indicated by horizontal lines. Effect estimates were adjusted for maternal education, ethnicity, pre-pregnancy BMI, maternal height, parity, energy intake (for DASH analysis only), cigarette smoking and alcohol consumption during pregnancy and child sex and age at measurement. E-DII, energy-adjusted Dietary Inflammatory Index; DASH, Dietary Approaches to Stop Hypertension; FFMI, fat-free mass index

Some potential sex-interactions were noted, especially for mid- and late-childhood FMI and FFMI outcomes (see Additional file 1: Table S18 and S19). In stratified analyses, the associations between a higher maternal pregnancy E-DII score and higher mid-childhood FMI, and a higher DASH score with lower mid-childhood FMI, were stronger in females (Fig. 2a) (all *P*-for-sex interactions < 0.10). In contrast, higher maternal pregnancy E-DII score was associated with lower [*β* (95% CI) − 0.10 (− 0.14, − 0.06) kg/m^2^], whereas higher DASH score was associated with higher [*β* (95% CI) 0.06 (0.02, 0.09) kg/m^2^], late-childhood FFMI in males (Fig. 2b) (all *P*-for-sex interactions < 0.10).

## Discussion

We observed that higher maternal dietary quality (higher DASH) during pregnancy was associated with a lower odds of late-childhood OWOB and lower FMI. In contrast, a more pro-inflammatory (higher E-DII) diet during pregnancy was associated with lower late-childhood FFMI. Furthermore, sex interactions were observed such that the associations of higher maternal DASH, lower E-DII and higher late-childhood FFMI were stronger in male offspring, while associations of lower maternal DASH, higher E-DII and higher mid-childhood FMI were stronger in female offspring. Contrary to our hypothesis, dietary score associations at different pregnancy stages were quite similar. Thus, the discussion of results will be based on the whole pregnancy period with the highest number of participants.

Previous studies showed that in US and Greek children (total *n* = 1566), higher maternal Mediterranean Diet Score (reflecting higher dietary quality) was associated with lower mid-childhood BMI *z*-score and SST [20]. In the Mothers and Infants LinKed for Health study (*n* = 354), higher maternal diet quality (higher score on Healthy Eating Index) from pregnancy through 3 months postpartum was associated with lower infant weight-for-length *z*-score from birth to 6 months and body fat % at 6 months [21]. In Project Viva (*n* = 992), a more pro-inflammatory diet (higher DII scores) during pregnancy was associated with higher mid-childhood (median age: 7.7 years) adiposity, e.g. FMI and SST, but attenuated after adjustment for covariates [22]. The Healthy Start Study (*n* = 1078) reported that higher DII scores in obese mothers were associated with increased neonatal fat mass [23]. Overall, results from our and these previous studies suggested beneficial influence of better maternal dietary quality and lower dietary inflammatory potential on offspring adiposity measures.

That maternal diet can influence offspring adiposity is biologically plausible. Maternal inflammation has been associated with higher offspring adiposity in both animal [16] and human [15] studies. One potential mechanism underpinning the association between overall healthier maternal diet and lower offspring adiposity could be lowered maternal inflammation due to high intakes of foods rich in anti-oxidants such as fruit and vegetables [60, 61]. However, our mutual adjustment analyses did not suggest that the influence of higher maternal DASH scores on childhood adiposity were mostly explained by maternal dietary inflammatory potential. Child dietary inflammatory potential also did not seem to meaningfully alter the maternal E-DII vs postnatal adiposity associations, suggesting that childhood diet/influence of shared familial diet is not a major mediator for our observations. An alternative mechanism could be maternal diet-induced modifications of epigenetic patterns in offspring. Individuals who were prenatally exposed to extreme maternal undernutrition demonstrated persistent epigenetic differences 6 decades later, as compared to their unexposed, same-sex siblings [62]. Milder in utero nutritional challenges such as lower maternal dietary quality (low adherence to Mediterranean diet) [63], carbohydrate amount [64] and quality [65] have also been shown to induce epigenetic differences in offspring at birth. Moreover, some of these epigenetic effects, e.g. greater methylation of RXRA chr9:136355885+, may be correlated with greater adiposity in later childhood [64].

Adipocyte proliferation is high during the first year of life and from age 9–14 years but remains low in between [64, 66]. Due to the availability of data in our constituent cohorts, early childhood adiposity measures were recorded at a mean age of 2.8 years. Thus, we might have missed the window to detect more apparent associations between maternal diet and early infancy adiposity. It is also possible that alterations of gene expression in offspring induced by maternal diets can present themselves only later in life. For example, in one animal study, increased expression of PPARα and CPT-1 (key genes in lipid and carbohydrate metabolism) was detected only in adult, but not neonatal, offspring of pregnant rat dams fed a protein-restricted diet [67]. However, these postulated mechanisms should be further investigated and confirmed in a human population with greater granularity of adiposity measurements.

Contrary to our expectation, higher E-DII score was associated with a lower odds of early childhood OWOB. The BMI curve in childhood is characterized by an initial increase until a peak at 1 year old, then dropping to a nadir around age 6 and starts rising again (a phenomenon termed adiposity rebound). However, there is variation of timing of adiposity rebound and children or adults who become obese have been reported as having an earlier adiposity rebound (around 3 years old) in early childhood [68]. Those with a normal adiposity rebound timing might thus have a higher BMI compared to early rebounders, a potential explanation of this unexpected result. This association was not present when a stricter cut-off for obesity (> 95th percentile) was used, indicating that the unexpected association was influenced by a population that is less morbid. Alternatively, this unexpected association could represent chance finding as it is unlikely that a pro-inflammatory maternal diet is protective against childhood OWOB.

While a very useful and relevant measure, a higher BMI may not only arise from greater body fat, but also from higher fat-free mass, making it an imperfect measure of adiposity [69, 70]. The differential magnitude and velocity of fat and fat-free mass accretion during different growth periods further complicates the interpretation of changes in childhood BMI over time [71, 72]. Our results suggest that dietary inflammatory potential and overall quality may influence different aspects of offspring body composition. For example, we observed that a higher E-DII score (pro-inflammatory maternal diet) tended to associate with a higher odds of late childhood OWOB and lower FFMI; a lower FFMI can reflect overall lower muscle or lean mass, which has been associated with a higher risk of metabolic syndrome in children [73, 74]. However, for our late-childhood FMI and FFMI outcomes, only two UK cohorts (SWS and ALSPAC) were included for our analysis. Therefore, the generalizability of our observations should be confirmed in further studies. Nonetheless, the sex-interaction noted for maternal dietary quality and inflammatory potential with FMI and FFMI during mid- and late-childhood in our study merits further investigation, as sexual dimorphism in body composition and epigenetic changes in response to early life factors is well-documented [75–79]. Difference in body composition between boys and girls accentuates during later childhood in response to hormonal changes and could be an explanation of our results [80].

The large sample size and substantial efforts spent in harmonizing and curating data across multiple studies are the major strengths of our study. To our knowledge, the current study represents the largest multi-centre collaborative effort in investigating the influence of maternal dietary quality and inflammatory potential on childhood adiposity outcomes.

However, some limitations are worth noting. Our study can mainly be generalized to European-born/White women in developed countries. Nonetheless, we did include studies from a range of geographical regions within Europe (Ireland, British Isles, Western and Eastern Continental Europe), in which some diversity in dietary intakes and sociodemographic characteristics was observed. Self-reported dietary data were used, which might have increased non-differential measurement errors that may bias results towards the null. Moreover, the FFQs were mainly validated for European-born/White women (e.g. in Gen R), potentially introducing heterogeneity and more measurement errors for non-European-born/non-White women. Indeed, in our analyses restricted to European-born/White population, the observed associations strengthened. We have applied a commonly used energy intake cut-off for pregnant women [54, 55] to exclude implausible dietary data. Other more conservative approaches such as specifically excluding misreporters (under- and over-reporters) of dietary intakes could be considered in future studies. Generation of E-DII and the DASH scores in our study might be subject to measurement errors as they are dependent on participants’ self reports and involved variable (20–28 out of 44 possible) dietary parameters, and 48.1–79.1% of the total FFQ food items, respectively. However, former studies have found a good predictive ability of E-DII with as few as 18 E-DII parameters [19], while each food component of the DASH score comprised at least five food items [36]. In some cohorts, children’s weights were self-reported rather than measured, potentially resulting in higher random measurement errors as it is unlikely that the errors depend on exposure. We did not find any consistent association with SST, probably due to challenges to obtain accurate skinfold measurements in young children, resulting in larger random measurement errors [81]. As with any observational study, the influence of residual confounding cannot be completely ruled out. For instance, education attainment categorization and subsequently confounding control was presumably less precise in this study due to the need to harmonize the variable across cohorts. Furthermore, although we have harmonized and adjusted for many essential covariates, future studies should also consider other potential covariates such as physical activity and mediators such as gestational weight gain and detailed data on breastfeeding duration. Finally, causality cannot be established without further complementary evidence.

## Conclusions

Our individual participant data meta-analysis within a large consortium suggests that pro-inflammatory, low-quality maternal pregnancy diets may adversely influence offspring adiposity and obesity risk, especially during late-childhood. Promoting overall healthy dietary pattern during pregnancy may have lifelong consequences for the offspring. Because most associations were observed at mid-childhood or later, future studies investigating in utero programming of childhood adiposity may benefit from a longer follow-up.

## Supplementary Information

**Additional file 1: Table S1-S19 and Fig. S1-S2**. **Table S1** Characteristics of study participants according to included studies. **Table S2** Characteristics of the cohorts in the ALPHABET consortium. **Table S3** Food parameters included for E-DII generation. **Table S4** Food items included for DASH score generation. **Fig. S1** Boxplots of E-DII scores in included studies. **Table S5** Availability of outcome measures. **Fig. S2** Scatterplots of DASH score against E-DII score in each study. **Table S6** Association between maternal E-DII and DASH scores (per 1-SD increase) and childhood OWOB- excluding non-European-born/non-White participants. **Table S7** Association between maternal E-DII and DASH scores (per 1-SD increase) and secondary childhood adiposity measures- excluding non-European-born/non-White participants. **Table S8** Association between maternal E-DII and DASH scores (per 1-SD increase) and childhood OWOB- excluding mothers with pregnancy complications. **Table S9** Association between maternal E-DII and DASH scores (per 1-SD increase) and secondary childhood adiposity measures- excluding mothers with pregnancy complications. **Table S10** Association between maternal E-DII and DASH scores (per 1-SD increase) and childhood OWOB- with mutual adjustment of E-DII and DASH. **Table S11** Association between maternal E-DII and DASH scores (per 1-SD increase) and secondary childhood adiposity measures- with mutual adjustment of E-DII and DASH. **Table S12** Association between maternal E-DII and DASH scores (per 1-SD increase) and childhood obesity (BMI z-score > 95th percentile). **Table S13** Association between maternal E-DII and DASH scores (per 1-SD increase) and childhood OWOB- with further adjustment of birthweight. **Table S14** Association between maternal E-DII and DASH scores (per 1-SD increase) and secondary childhood adiposity measures- with further adjustment of birthweight. **Table S15** Association between maternal E-DII and DASH scores (per 1-SD increase) and childhood OWOB- with further adjustment of gestational age. **Table S16** Association between maternal E-DII and DASH scores (per 1-SD increase) and secondary childhood adiposity measures- with further adjustment of gestational age. **Table S17** Association between maternal E-DII (per 1-SD increase) and late-childhood OWOB and adiposity measures- with and without further adjustment for child E-DII score in cohorts with child E-DII data. **Table S18** Pooled *P*-values for sex-interaction between maternal E-DII and DASH score and offspring adiposity outcomes. **Table S19** Stratified estimates for other sex-interactions between maternal E-DII and DASH scores and offspring adiposity outcomes (all P-interactions < 0.10).

## Data Availability

The data that support the findings of this study are available from the individual studies constituting this consortium but restrictions apply to the availability of these data, which were used under license for the current study, and so are not publicly available. Data are however available from the authors upon reasonable request and with permission from the executive committee in charge of the respective studies.

## Abbreviations

ALSPAC: The Avon Longitudinal Study of Parents and Children
BMI: Body mass index
DASH: Dietary Approaches to Stop Hypertension
DOHaD: Developmental Origins of Health and Diseases
EDEN: The study on the pre- and early postnatal determinants of child health and development
E-DII: Energy-adjusted Dietary Inflammatory Index
FFMI: Fat-free mass index
FFQ: Food frequency questionnaire
FMI: Fat mass index
Generation R: The Generation R study
Lifeways: The Lifeways Cross-Generation Cohort Study
OWOB: Overweight and obesity
REPRO_PL: The Polish Mother and Child Cohort
ROLO: The Randomised cOntrol trial of LOw glycaemic index diet during pregnancy study
SST: Sum of skinfold thickness
SWS: Southampton Women’s Survey
WHO: World Health Organization
