# Supplementary material for: Maternal dietary quality, inflammatory potential and childhood adiposity: an individual participant data pooled analysis of seven European cohorts in the ALPHABET consortium
Source: BMC Med. 2021 Feb 22;19:33. doi: 10.1186/s12916-021-01908-7 (PMC7898733; doi:10.1186/s12916-021-01908-7)
Supplement: Supplementary file 1 — Additional file 1: Table S1-S19 and Fig. S1-S2. Table S1 Characteristics of study participants according to included studies. Table S2 Characteristics of the cohorts in the ALPHABET consortium. Table S3 Food parameters included for E-DII generation. Table S4 Food items included for DASH score generation. Fig. S1 Boxplots of E-DII scores in included studies. Table S5 Availability of outcome measures. Fig. S2 Scatterplots of DASH score against E-DII score in each study. Table S6 Association between maternal E-DII and DASH scores (per 1-SD increase) and childhood OWOB- excluding non-European-born/non-White participants. Table S7 Association between maternal E-DII and DASH scores (per 1-SD increase) and secondary childhood adiposity measures- excluding non-European-born/non-White participants. Table S8 Association between maternal E-DII and DASH scores (per 1-SD increase) and childhood OWOB- excluding mothers with pregnancy complications. Table S9 Association between maternal E-DII and DASH scores (per 1-SD increase) and secondary childhood adiposity measures- excluding mothers with pregnancy complications. Table S10 Association between maternal E-DII and DASH scores (per 1-SD increase) and childhood OWOB- with mutual adjustment of E-DII and DASH. Table S11 Association between maternal E-DII and DASH scores (per 1-SD increase) and secondary childhood adiposity measures- with mutual adjustment of E-DII and DASH. Table S12 Association between maternal E-DII and DASH scores (per 1-SD increase) and childhood obesity (BMI z-score > 95th percentile). Table S13 Association between maternal E-DII and DASH scores (per 1-SD increase) and childhood OWOB- with further adjustment of birthweight. Table S14 Association between maternal E-DII and DASH scores (per 1-SD increase) and secondary childhood adiposity measures- with further adjustment of birthweight. Table S15 Association between maternal E-DII and DASH scores (per 1-SD increase) and childhood OWOB- with further adjustment of [file 12916_2021_1908_MOESM1_ESM.docx]

**Maternal dietary quality, inflammatory potential and childhood adiposity: an individual participant data pooled analysis of seven European cohorts in the ALPHABET consortium**

Ling-Wei Chen, Adrien M Aubert, Nitin Shivappa, Jonathan Y Bernard, Sara M Mensink-Bout, Aisling A Geraghty, John Mehegan, Matthew Suderman, Kinga Polanska, Wojciech Hanke, Agnieszka Jankowska, Caroline L Relton, Sarah R Crozier, Nicholas C Harvey, Cyrus Cooper, Mark Hanson, Keith M Godfrey, Romy Gaillard, Liesbeth Duijts, Barbara Heude, James R Hébert, Fionnuala M McAuliffe, Cecily C Kelleher, Catherine M Phillips

**Description of additional files**
**Additional File 1: Table S1** Characteristics of study participants according to included studies

**Additional File 1: Table S2** Characteristics of the cohorts in the ALPHABET consortium

**Additional File 1: Table S3** Food parameters included for E-DII generation

**Additional File 1: Table S4** Food items included for DASH score generation

**Additional File 1: Fig. S1** Boxplots of E-DII scores in included studies

**Additional File 1: Table S5** Availability of outcome measures

**Additional File 1: Fig. S2** Scatterplots of DASH score against E-DII score in each study

**Additional File 1: Table S6** Association between maternal E-DII and DASH scores (per 1-SD increase) and childhood OWOB- excluding non-European-born/non-White participants

**Additional File 1: Table S7** Association between maternal E-DII and DASH scores (per 1-SD increase) and secondary childhood adiposity measures- excluding non-European-born/non-White participants

**Additional File 1: Table S8** Association between maternal E-DII and DASH scores (per 1-SD increase) and childhood OWOB- excluding mothers with pregnancy complications

**Additional File 1: Table S9** Association between maternal E-DII and DASH scores (per 1-SD increase) and secondary childhood adiposity measures- excluding mothers with pregnancy complications

**Additional File 1: Table S10** Association between maternal E-DII and DASH scores (per 1-SD increase) and childhood OWOB- with mutual adjustment of E-DII and DASH

**Additional File 1: Table S11** Association between maternal E-DII and DASH scores (per 1-SD increase) and secondary childhood adiposity measures- with mutual adjustment of E-DII and DASH

**Additional File 1: Table S12** Association between maternal E-DII and DASH scores (per 1-SD increase) and childhood obesity (BMI z-score >95^th^ percentile)

**Additional File 1: Table S13** Association between maternal E-DII and DASH scores (per 1-SD increase) and childhood OWOB- with further adjustment of birthweight

**Additional File 1: Table S14** Association between maternal E-DII and DASH scores (per 1-SD increase) and secondary childhood adiposity measures- with further adjustment of birthweight

**Additional File 1: Table S15** Association between maternal E-DII and DASH scores (per 1-SD increase) and childhood OWOB- with further adjustment of gestational age

**Additional File 1: Table S16** Association between maternal E-DII and DASH scores (per 1-SD increase) and secondary childhood adiposity measures- with further adjustment of gestational age

**Additional File 1: Table S17** Association between maternal E-DII (per 1-SD increase) and late-childhood OWOB and adiposity measures- with and without further adjustment for child E-DII score in cohorts with child E-DII data

**Additional File 1: Table S18** Pooled *P*-values for sex-interaction between maternal E-DII and DASH score and offspring adiposity outcomes

**Additional File 1: Table S19** Stratified estimates for other sex-interactions between maternal E-DII and DASH scores and offspring adiposity outcomes (all *P*-interactions <0.10)

**Additional File 1: Table S1** Characteristics of study participants according to included studies [27-35]*

| **Cohort name** | ALPHABET overall | ALSPAC† | EDEN | GEN R | Lifeways | REPRO_PL | ROLO | SWS | ***P***-value‡ |
| --- | --- | --- | --- | --- | --- | --- | --- | --- | --- |
| **Max number included** | 16295 | 6507 | 1256 | 5079 | 546 | 380 | 386 | 2141 |  |
| Pre-pregnancy E-DII, mean (SD) | 0.0 (1.7) | - | 0.4 (1.7) | - | - | - | - | -0.2 (1.6) | <0.001 |
| Pregnancy E-DII, mean (SD) | 0.1 (1.6) | 0.4 (1.8) | 0.8 (1.6) | -0.4 (1.1) | 0.1 (1.6) | -1.2 (1.5) | 0.1 (1.7) | 0.4 (1.3) | <0.001 |
| Pre-pregnancy DASH, mean (SD) | 24.3 (4.3) | - | 24.3 (4.1) | - | - | - | - | 24.3 (4.4) | 0.84 |
| Pregnancy DASH, mean (SD) | 24.3 (4.2) | 24.3 (4.0) | 24.2 (4.1) | 24.2 (4.5) | 24.2 (4.5) | 24.1 (4.3) | 23.9 (4.2) | 24.3 (3.9) | 0.46 |
| Maternal age, y, mean (SD) | 30.2 (4.6) | 29.6 (4.5) | 30.0 (4.7) | 30.6 (4.9) | 31.2 (5.5) | 29.3 (4.1) | 33.1 (3.9) | 30.8 (3.8) | <0.001 |
| Pre-pregnancy BMI, kg/m^2^, mean (SD) | 23.4 (4.2) | 22.5 (4.0) | 23.2 (4.4) | 23.5 (3.9) | 23.8 (3.5) | 22.4 (3.7) | 26.2 (4.5) | 25.3 (4.7) | <0.001 |
| Maternal height, cm, mean (SD) | 165.4 (7.0) | 164.2 (6.5) | 163.6 (6.1) | 168.3 (7.3) | 163.6 (6.4) | 166.6 (5.7) | 166.1 (6.4) | 163.2 (6.5) | <0.001 |
| *Maternal education level* |  |  |  |  |  |  |  |  | <0.001 |
| Low | 2223 (13.6%) | 874 (13.4%) | 51 (4.1%) | 354 (7.0%) | 85 (15.6%) | 24 (6.3%) | 2 (0.5%) | 833 (38.9%) |  |
| Medium | 8195 (50.3%) | 4614 (70.9%) | 214 (17.0%) | 2119 (41.7%) | 273 (50.0%) | 99 (26.1%) | 73 (18.9%) | 803 (37.5%) |  |
| High | 5877 (36.1%) | 1019 (15.7%) | 991 (78.9%) | 2606 (51.3%) | 188 (34.4%) | 257 (67.6%) | 311 (80.6%) | 505 (23.6%) |  |
| *Maternal ethnicity*§ |  |  |  |  |  |  |  |  | <0.001 |
| European-born/White | 14437 (88.6%) | 6393 (98.2%) | 1226 (97.6%) | 3449 (67.9%) | 546 (100.0%) | 380 (100.0%) | 382 (99.0%) | 2061 (96.3%) |  |
| Non-European-born/non-White | 1858 (11.4%) | 114 (1.8%) | 30 (2.4%) | 1630 (32.1%) | 0 (0.0%) | 0 (0.0%) | 4 (1.0%) | 80 (3.7%) |  |
| *Parity* |  |  |  |  |  |  |  |  | <0.001 |
| Nulliparous | 8097 (49.7%) | 2984 (45.9%) | 583 (46.4%) | 2976 (58.6%) | 222 (40.7%) | 226 (59.5%) | 0 (0.0%)\|\| | 1106 (51.7%) |  |
| Non-nulliparous | 8198 (50.3%) | 3523 (54.1%) | 673 (53.6%) | 2103 (41.4%) | 324 (59.3%) | 154 (40.5%) | 386 (100.0%) | 1035 (48.3%) |  |
| *Cigarette smoking during pregnancy* |  |  |  |  |  |  |  |  | <0.001 |
| Never | 9938 (61.0%) | 3681 (56.6%) | 979 (77.9%) | 3174 (62.5%) | 237 (43.4%) | 249 (65.5%) | 375 (97.2%) | 1243 (58.1%) |  |
| Ever | 3874 (23.8%) | 1673 (25.7%) | 3 (0.2%) | 1326 (26.1%) | 175 (32.1%) | 86 (22.6%) | 0 (0.0%) | 611 (28.5%) |  |
| Current | 2483 (15.2%) | 1153 (17.7%) | 274 (21.8%) | 579 (11.4%) | 134 (24.5%) | 45 (11.8%) | 11 (2.8%) | 287 (13.4%) |  |
| *Alcohol consumption during pregnancy* |  |  |  |  |  |  |  |  | <0.001 |
| No | 7587 (46.6%) | 2811 (43.2%) | 913 (72.7%) | 2810 (55.3%) | 157 (28.8%) | 346 (91.1%) | 107 (27.7%) | 443 (20.7%) |  |
| Yes | 8708 (53.4%) | 3696 (56.8%) | 343 (27.3%) | 2269 (44.7%) | 389 (71.2%) | 34 (8.9%) | 279 (72.3%) | 1698 (79.3%) |  |
| *Child sex* |  |  |  |  |  |  |  |  | 0.48 |
| Male | 8182 (50.2%) | 3239 (49.8%) | 660 (52.5%) | 2527 (49.8%) | 272 (49.8%) | 186 (48.9%) | 194 (50.3%) | 1104 (51.6%) |  |
| Female | 8113 (49.8%) | 3268 (50.2%) | 596 (47.5%) | 2552 (50.2%) | 274 (50.2%) | 194 (51.1%) | 192 (49.7%) | 1037 (48.4%) |  |
|  |  |  |  |  |  |  |  |  |  |
| Primary outcomes |  |  |  |  |  |  |  |  |  |
| Early-childhood OWOB |  |  |  |  |  |  |  |  | <0.001 |
| No | 5612 (85.1%) | 778 (78.4%) | 1016 (91.9%) | 1703 (85.8%) | - | 197 (90.4%) | 241 (88.0%) | 1677 (83.1%) |  |
| Yes | 981 (14.9%) | 214 (21.6%) | 90 (8.1%) | 281 (14.2%) | - | 21 (9.6%) | 33 (12.0%) | 342 (16.9%) |  |
| Mid-childhood OWOB |  |  |  |  |  |  |  |  | <0.001 |
| No | 7767 (85.1%) | 773 (86.8%) | 967 (93.6%) | 3810 (84.0%) | 381 (76.2%) | 206 (82.1%) | 259 (82.7%) | 1371 (85.4%) |  |
| Yes | 1363 (14.9%) | 118 (13.2%) | 66 (6.4%) | 727 (16.0%) | 119 (23.8%) | 45 (17.9%) | 54 (17.3%) | 234 (14.6%) |  |
| Late-childhood OWOB |  |  |  |  |  |  |  |  | <0.001 |
| No | 10277 (85.2%) | 5150 (83.4%) | 582 (93.0%) | 3424 (86.5%) | 207 (80.9%) | - | - | 914 (87.8%) |  |
| Yes | 1780 (14.8%) | 1025 (16.6%) | 44 (7.0%) | 535 (13.5%) | 49 (19.1%) | - | - | 127 (12.2%) |  |

*Values were mean (SD) for continuous variables or n (%) for categorical variables.

†Pregnant women resident in Avon, UK with expected dates of delivery 1st April 1991 to 31st December 1992 were invited to take part in the study. The initial number of pregnancies enrolled is 14,541 (for these at least one questionnaire has been returned or a “Children in Focus” clinic had been attended by 19/07/99). Of these initial pregnancies, there were a total of 14,676 foetuses, resulting in 14,062 live births and 13,988 children who were alive at 1 year of age.

‡*P*-values were obtained from one-way ANOVA for continuous variables and chi-square test for categorical variables.

§For EDEN, maternal ethnicity was proxied by place of birth using the question ‘Are you born in Europe? Outside Europe?’, because specific question on ethnicity is not allowed in France.

||ROLO’s mothers are all second gradiva (recruitment criteria).

OWOB, overweight and obesity

**Additional File 1: Table S2** Characteristics of the cohorts in the ALPHABET consortium*

| **Cohort** | **ALSPAC** | **EDEN** | | **Generation R** | **Lifeways** | **REPRO_PL** | **ROLO** | **SWS** | | |
| --- | --- | --- | --- | --- | --- | --- | --- | --- | --- | --- |
| **Number of Recruited Women** | 14,541 | 2002 | | 9778 | 1132 | 1451 | 759 | 12,583 | | |
| Women age eligibility | No age limit | 18 and over | | No age limit | No age limit | No age limit | 18 and over | 20–34 years | | |
| Study type | Mother-child cohort | Mother-child cohort | | Pregnancy-child cohort | Mother-child cohort | Mother-child cohort | Randomised control trial | Pre-pregnancy and pregnancy-child cohort | | |
| Period of inclusion | 1990–1992 | 2003–2006 | | 2002–2006 | 2001–2003 | 2007–2011 | 2007–2011 | 1998–2002 | | |
| Location (specific cities) | England (Bristol) | France (Multicentre) | | The Netherlands (Rotterdam) | Republic of Ireland (Multicentre) | Poland (Multicentre) | Republic of Ireland (Dublin) | England (Southampton) | | |
| Assessment types | Non-quantitative FFQ | Semi-quantitative FFQ | | Semi-quantitative FFQ | Semi-quantitative FFQ | Non-quantitative FFQ | Semi-quantitative FFQ | Non-quantitative FFQ | | |
| Period of FFQ assessment | Around 32 WG | 24–28 WG | Birth | <24 WG | 12–16 WG | 20–24 WG | ≤28 WG | PP | 11 WG | 34 WG |
| FFQ window period | LP | PP | LP | EP | EP | EP | EP | PP | EP | LP |
| Number of women with validated FFQ ^1^ | 11,965 | 1964 | 1849 | 6402^2^ | 1121 | 1314 | 631 | 3156^3^ | 2270 | 2649 |
| Mode of FFQ assessment | Self-reported | Self-reported | | Self-reported | Self-reported | Self-reported | Self-reported | Nurse administered | | |

FFQ: Food Frequency Questionnaire. WG: weeks of gestation. PP: pre-pregnancy, EP: early pregnancy, LP: late pregnancy. ^1^ Finally included in this study. ^2^ Generation R used a caloric cut-off to exclude women with caloric intakes <500 kcal or >3500 kcal (all other cohorts did not make any exclusion based on energy before deriving the DASH score). ^3^ 12,572 women answered the FFQ at PP period but only 3158 women went on to have live singleton birth within the study.

*Reproduced in entirety from reference below, which was published in open access journal granting open access Creative Common CC BY license, meaning any part of the article may be reused without permission provided that the original article is clearly cited.

Aubert AM, Forhan A, de Lauzon-Guillain B, Chen L-W, Polanska K, Hanke W, et al. Deriving the Dietary Approaches to Stop Hypertension (DASH) Score in Women from Seven Pregnancy Cohorts from the European ALPHABET Consortium. Nutrients. 2019;11:2706.

**Additional File 1: Table S3** Food parameters included for E-DII generation

| **Food parameters for E-DII generation** | **ALSPAC** | **EDEN** | **Gen R** | **Lifeways** | **REPRO_PL** | **ROLO** | **SWS** |
| --- | --- | --- | --- | --- | --- | --- | --- |
| Beta Carotene | ✓ | ✓ | - | ✓ | ✓ | ✓ | ✓ |
| Folic Acid | ✓ | ✓ | - | ✓ | ✓ | ✓ | ✓ |
| Vitamin A | ✓ | ✓ | - | ✓ | ✓ | ✓ | ✓ |
| Alcohol | ✓ | ✓ | ✓ | ✓ | ✓ | ✓ | ✓ |
| Carbohydrate | ✓ | ✓ | ✓ | ✓ | ✓ | ✓ | ✓ |
| Cholesterol | ✓ | ✓ | ✓ | ✓ | ✓ | ✓ | ✓ |
| Fat | ✓ | ✓ | ✓ | ✓ | ✓ | ✓ | ✓ |
| Fiber | ✓ | ✓ | ✓ | ✓ | ✓ | ✓ | ✓ |
| Iron | ✓ | ✓ | ✓ | ✓ | ✓ | ✓ | ✓ |
| Magnesium | ✓ | ✓ | ✓ | ✓ | ✓ | ✓ | ✓ |
| MUFA | ✓ | ✓ | ✓ | ✓ | ✓ | ✓ | ✓ |
| Niacin | ✓ | ✓ | - | ✓ | ✓ | ✓ | ✓ |
| Protein | ✓ | ✓ | ✓ | ✓ | ✓ | ✓ | ✓ |
| PUFA | ✓ | ✓ | ✓ | ✓ | ✓ | ✓ | ✓ |
| Riboflavin | ✓ | ✓ | ✓ | ✓ | ✓ | ✓ | ✓ |
| Saturated fat | ✓ | ✓ | ✓ | ✓ | ✓ | ✓ | ✓ |
| Selenium | ✓ | - | - | ✓ | ✓ | ✓ | - |
| Thiamin | ✓ | ✓ | - | ✓ | ✓ | ✓ | - |
| Vitamin B 12 | ✓ | ✓ | - | ✓ | ✓ | ✓ | ✓ |
| Vitamin B 6 | ✓ | ✓ | ✓ | ✓ | ✓ | ✓ | ✓ |
| Vitamin C | ✓ | ✓ | ✓ | ✓ | ✓ | ✓ | ✓ |
| Vitamin D | ✓ | ✓ | - | ✓ | ✓ | ✓ | ✓ |
| Vitamin E | ✓ | ✓ | - | ✓ | ✓ | ✓ | ✓ |
| Zinc | ✓ | - | ✓ | ✓ | ✓ | ✓ | ✓ |
| Garlic | - | - | ✓ | ✓ | - | ✓ | - |
| Onion (in grams) | - | - | ✓ | ✓ | - | ✓ | ✓ |
| Tea (in grams) | ✓ | ✓ | ✓ | ✓ | ✓ | ✓ | ✓ |
| Caffeine | ✓ | - | ✓ | ✓ | ✓ | ✓ | - |
| Omega 3 | ✓ | ✓ | - | - | ✓ | - | - |
| Omega 6 | - | ✓ | ✓ | - | ✓ | - | - |
| Trans Fat | ✓ | - | - | - | - | - | - |
| **Total parameters** | **28** | **25** | **20** | **28** | **28** | **28** | **24** |

PUFA, polyunsaturated fatty acids; MUFA, monounsaturated fatty acid

| **Cohort** | **ALSPAC** | **EDEN** | **Gen R** | **Lifeways** | **REPRO_PL** | **ROLO** | **SWS** |
| --- | --- | --- | --- | --- | --- | --- | --- |
| Total of FFQ food items selected for the DASH | 34 | 65 | 136 | 85 | 36 | 85 | 58 |
| % FFQ items selected/total food items without alcohol | 79.1% | 50.0% | 48.1% | 55.2% | 58.1% | 55.2% | 58.6% |
| **Food components with higher intakes recommended** | | | | | | | |
| Total grains | 7 | 7 | 20 | 14 | 5 | 14 | 8 |
| Vegetables (excluding potatoes and condiments) | 5 | 16 | 33 | 24 | 12 | 24 | 16 |
| Fruits | 3 | 12 | 20 | 13 | 10 | 13 | 12 |
| Non-full-fat dairy products | 3 | 6 | 18 | 7 | 2 | 7 | 5 |
| Nuts, seeds, legumes | 7 | 4 | 14 | 5 | 2 | 5 | 2 |
| **Food components with lower intakes recommended** | | | | | | | |
| Red and Processed meat | 4 | 12 | 20 | 17 | 4 | 17 | 10 |
| Sugar-sweetened beverages, sweets, and added sugars | 5 | 8 | 11 | 5 | 1 | 5 | 5 |
| Sodium | Available in grams/day | Available in grams/day | Available in grams/day | Available in grams/day | Available in grams/day | Available in grams/day | Available in grams/day |

**Additional File 1: Table S4** Food items included for DASH score generation

FFQ: Food frequency questionnaire. E-DII: Energy-adjusted Dietary Inflammatory Index. DASH: Dietary Approaches to Stop Hypertension

**Additional File 1: Fig. S1** Boxplots of E-DII scores in included studies

E-DII: Energy-adjusted Dietary Inflammatory Index. DASH: Dietary Approaches to Stop Hypertension

**Additional File 1: Table S5** Availability of outcome measures

| Cohort | **Early** | | | |  | **Mid** | | | |  | **Late** | | | |
| --- | --- | --- | --- | --- | --- | --- | --- | --- | --- | --- | --- | --- | --- | --- |
|  | BMI | SST | FMI | FFMI |  | BMI | SST | FMI^*^ | FFMI^*^ |  | BMI | SST | FMI^*^ | FFMI^*^ |
| ALSPAC | ✓ | - | - | - |  | ✓ | ✓ | - | - |  | ✓ | - | BIA | BIA |
| EDEN | ✓ | ✓ | - | - |  | ✓ | ✓ | BIA | BIA |  | ✓ | - | - | - |
| GEN R | ✓ | - | - | - |  | ✓ | - | - | - |  | ✓ | - | - | - |
| Lifeways | - | - | - | - |  | ✓ | - | - | - |  | ✓ | - | - | - |
| REPRO_PL | ✓ | - | - | - |  | ✓ | ✓ | BIA | BIA |  | - | - | - | - |
| ROLO | ✓ | - | - | - |  | ✓ | ✓ | BIA | BIA |  | - | - | - | - |
| SWS | ✓ | ✓ | - | - |  | ✓ | ✓ | DXA | DXA |  | ✓ | ✓ | DXA | DXA |

^*^Child fat mass and fat free mass in the current study were measured using bioelectrical impedance analysis (BIA), except for SWS, which was measured using Dual-energy X-ray absorptiometry (DXA).

BMI, body mass index; SST, sum-of-skinfold-thickness; FMI, fat-mass-index; FFMI, fat-free-mass-index

ALSPAC, the Avon Longitudinal Study of Parents and Children; EDEN, the study on the pre- and early postnatal determinants of child health and development; GEN R, The Generation R Study; Lifeways, Lifeways Cross-Generation Cohort Study; REPRO_PL, the Polish Mother and Child Cohort; ROLO, the Randomised cOntrol trial of LOw glycaemic index diet during pregnancy; SWS, the Southampton Women’s Survey

**Additional File 1: Fig. S2** Scatterplots of DASH scores against E-DII scores in each study

E-DII: Energy-adjusted Dietary Inflammatory Index; DASH: Dietary Approaches to Stop Hypertension

**Additional File 1: Table S6** Association between maternal E-DII and DASH scores (per 1-SD increase) and childhood OWOB- excluding non-European-born/non-White participants

|  | Early-childhood |  | Mid-childhood |  |  | Late-childhood |  |
| --- | --- | --- | --- | --- | --- | --- | --- |
|  | OR (95%CI) | *I^2^ (%)* | OR (95%CI) | *I^2^ (%)* |  | OR (95%CI) | *I^2^ (%)* |
| **E-DII** |  |  |  |  |  |  |  |
| *Pre* | 0.93 (0.84, 1.04) | 0 | 1.04 (0.91, 1.18) | 0 |  | 0.96 (0.80, 1.15) | 0 |
| Np/Nc | 3020/2 |  | 2557/2 |  |  | 1625/2 |  |
| *Preg* | 0.95 (0.88, 1.04) | 0 | 0.97 (0.90, 1.04) | 0 |  | 1.10 (0.99, 1.23) | 44 |
| Np/Nc | 5393/6 |  | 7232/7 |  |  | 10554/5 |  |
| *Early* | 0.999 (0.90, 1.11) | 0 | 0.98 (0.90, 1.06) | 0 |  | 1.14 (1.02, 1.28)* | 0 |
| Np/Nc | 3434/4 |  | 5436/5 |  |  | 3920/3 |  |
| *Late* | 0.91 (0.83, 0.999)* | 0 | 0.98 (0.85, 1.12) | 28 |  | 1.08 (0.94, 1.24) | 40 |
| Np/Nc | 3913/3 |  | 3344/3 |  |  | 7642/3 |  |
|  |  |  |  |  |  |  |  |
| **DASH** |  |  |  |  |  |  |  |
| *Pre* | 0.98 (0.76, 1.26) | 73 | 0.91 (0.79, 1.04) | 0 |  | 0.97 (0.80, 1.16) | 0 |
| Np/Nc | 3020/2 |  | 2557/2 |  |  | 1625/2 |  |
| *Preg* | 0.95 (0.87, 1.04) | 0 | 0.99 (0.92, 1.07) | 0 |  | 0.90 (0.85, 0.96)** | 0 |
| Np/Nc | 5393/6 |  | 7232/7 |  |  | 10554/5 |  |
| *Early* | 0.95 (0.85, 1.06) | 0 | 1.01 (0.92, 1.11) | 9 |  | 0.84 (0.75, 0.94)** | 0 |
| Np/Nc | 3424/4 |  | 5436/5 |  |  | 3920/3 |  |
| *Late* | 1.01 (0.91, 1.11) | 0 | 0.95 (0.85, 1.07) | 0 |  | 0.91 (0.85, 0.98)* | 0 |
| Np/Nc | 3913/3 |  | 3344/3 |  |  | 7642/3 |  |

Values are adjusted pooled effect estimates [OR (95% CI)] expressed for a 1-SD increment in dietary scores, heterogeneity measure (*I*^2^), and number of participants and studies included (Np/Nc) across different outcomes and conception periods, as labelled. Effect estimates were adjusted for maternal education, ethnicity, pre-pregnancy BMI, maternal height, parity, energy intake (for DASH analysis only), cigarette smoking and alcohol consumption during pregnancy, and (intrinsically adjusted for the outcome) child sex and age at measurement.

E-DII, energy-adjusted Dietary Inflammatory Index; DASH, Dietary Approaches to Stop Hypertension; OWOB, overweight and obesity; *I*^2^, *I*-squared; Pre, pre-pregnancy; Preg, pregnancy; Early, early pregnancy; Late, late pregnancy; Np, number of participants included; Nc, number of cohorts included.

**P*<0.05, ***P*<0.01

**Additional File 1: Table S7** Association between maternal E-DII and DASH scores (per 1-SD increase) and secondary childhood adiposity measures- excluding non-European-born/non-White participants

|  | Early-childhood  (2.8 ± 0.3 year) | |  | Mid-childhood  (6.1 ± 0.6 y) | | | | | |  | Late-childhood  (10.6 ± 1.2 y) | | | | | |
| --- | --- | --- | --- | --- | --- | --- | --- | --- | --- | --- | --- | --- | --- | --- | --- | --- |
|  | SST, mm |  |  | SST, mm |  | FMI, kg/m^2^ |  | FFMI, kg/m^2^ |  |  | SST, mm |  | FMI, kg/m^2^ |  | FFMI, kg/m^2^ |  |
|  | β (95%CI) | *I^2^ (%)* |  | β (95%CI) | *I^2^ (%)* | β (95%CI) | *I^2^ (%)* | β (95%CI) | *I^2^ (%)* |  | β (95%CI) | *I^2^ (%)* | β (95%CI) | *I^2^ (%)* | β (95%CI) | *I^2^ (%)* |
| **E-DII** |  |  |  |  |  |  |  |  |  |  |  |  |  |  |  |  |
| *Pre* | -0.09 (-0.21, 0.03) | 0 |  | 0.01 (-0.26, 0.28) | 52 | 0.01 (-0.03, 0.05) | 0 | -0.04 (-0.08, 0.01) | 0 |  | -0.17 (-0.76, 0.42) | - | 0.03 (-0.10, 0.16) | - | -0.04 (-0.11, 0.04) | - |
| Np/Nc | 2861/2 |  |  | 3279/3 |  | 1987/2 |  | 1990/2 |  |  | 993/1 |  | 823/1 |  | 823/1 |  |
| *Preg* | -0.01 (-0.15, 0.12) | 12 |  | 0.08 (-0.16, 0.31) | 29 | 0.03 (-0.03, 0.08) | 26 | -0.06 (-0.16, 0.03) | 67* |  | 0.36 (-0.29, 1.01) | - | 0.03 (-0.03, 0.09) | 0 | -0.05 (-0.08, -0.02)*** | 0 |
| Np/Nc | 2676/3 |  |  | 3114/4 |  | 2024/4 |  | 2027/4 |  |  | 760/1 |  | 6617/2 |  | 6617/2 |  |
| *Early* | 0.05 (-0.12, 0.23) | 0 |  | -0.02 (-0.29, 0.25) | 0 | 0.01 (-0.11, 0.13) | 43 | -0.13 (-0.33, 0.06) | 77* |  | 0.18 (-0.44, 0.81) | - | 0.03 (-0.10, 0.16) | - | -0.05 (-0.13, 0.03) | - |
| Np/Nc | 1668/2 |  |  | 1475/2 |  | 1103/3 |  | 1102/3 |  |  | 815/1 |  | 684/1 |  | 784/1 |  |
| *Late* | -0.05 (-0.17, 0.08) | 0 |  | 0.15 (-0.08, 0.37) | 20 | 0.03 (-0.02, 0.08) | 24 | 0.001 (-0.04, 0.05) | 0 |  | 0.30 (-0.29, 0.90) | - | 0.04 (-0.03, 0.11) | 9 | -0.05 (-0.08, -0.02)*** | 9 |
| Np/Nc | 2785/2 |  |  | 3096/3 |  | 1934/2 |  | 1937/2 |  |  | 928/1 |  | 6756/2 |  | 6756/2 |  |
|  |  |  |  |  |  |  |  |  |  |  |  |  |  |  |  |  |
| **DASH** |  |  |  |  |  |  |  |  |  |  |  |  |  |  |  |  |
| *Pre* | -0.04 (-0.22, 0.14) | 50 |  | -0.20 (-0.42, 0.01) | 24 | -0.04 (-0.08, -0.003)* | 0 | 0.03 (-0.01, 0.08) | 0 |  | -0.19 (-0.78, 0.41) | - | -0.08 (-0.21, 0.05) | - | 0.05 (-0.03, 0.12) | - |
| Np/Nc | 2861/2 |  |  | 2462/2 |  | 1987/2 |  | 1990/2 |  |  | 993/1 |  | 823/1 |  | 823/1 |  |
| *Preg* | -0.06 (-0.19, 0.07) | 0 |  | -0.02 (-0.30, 0.26) | 42 | -0.02 (-0.06, 0.02) | 0 | 0.02 (-0.03, 0.06) | 0 |  | -0.49 (-1.16, 0.18) | - | -0.09 (-0.15, -0.03)** | 0 | 0.03 (-0.002, 0.06) | 0 |
| Np/Nc | 2676/3 |  |  | 3114/4 |  | 2024/4 |  | 2027/4 |  |  | 760/1 |  | 6617/2 |  | 6617/2 |  |
| *Early* | -0.09 (-0.27, 0.10) | 0 |  | 0.10 (-0.41, 0.62) | 68 | -0.07 (-0.15, 0.01) | 0 | 0.04 (-0.02, 0.10) | 0 |  | -0.53 (-1.17, 0.12) | - | -0.10 (-0.23, 0.03) | - | 0.06 (-0.03, 0.14) | - |
| Np/Nc | 1668/2 |  |  | 1475/2 |  | 1103/3 |  | 1102/3 |  |  | 815/1 |  | 684/1 |  | 684/1 |  |
| *Late* | -0.02 (-0.15, 0.11) | 0 |  | -0.08 (-0.26, 0.10) | 0 | -0.01 (-0.05, 0.03) | 0 | 0.00 (-0.05, 0.05) | 0 |  | -0.30 (-0.91, 0.30) | - | -0.08 (-0.14, -0.02)* | 0 | 0.03 (-0.003, 0.05) | 0 |
| Np/Nc | 2785/2 |  |  | 3096/3 |  | 1934/2 |  | 1937/2 |  |  | 938/1 |  | 6756/2 |  | 6756/2 |  |

Values are adjusted pooled effect estimates [β (95% CI)] expressed for a 1-SD increment in dietary scores, heterogeneity measure (*I*^2^), and number of participants and studies included (Np/Nc) across different outcomes and conception periods, as labelled. Effect estimates were adjusted for maternal education, ethnicity, pre-pregnancy BMI, maternal height, parity, energy intake (for DASH analysis only), cigarette smoking and alcohol consumption during pregnancy, and child sex and age at measurement.

E-DII, energy-adjusted Dietary Inflammatory Index; DASH, Dietary Approaches to Stop Hypertension; SST, sum of skinfold thickness; FMI, fat mass index; FFMI, fat free mass index; *I*^2^, *I*-squared; Pre, pre-pregnancy; Preg, pregnancy; Early, early pregnancy; Late, late pregnancy; Np, number of participants included; Nc, number of cohorts included.

**P*<0.05, ***P*<0.01

**Additional File 1: Table S8** Association between maternal E-DII and DASH scores (per 1-SD increase) and childhood OWOB- excluding mothers with pregnancy complications

|  | Early-childhood |  | Mid-childhood |  |  | Late-childhood |  |
| --- | --- | --- | --- | --- | --- | --- | --- |
|  | OR (95%CI) | *I^2^ (%)* | OR (95%CI) | *I^2^ (%)* |  | OR (95%CI) | *I^2^ (%)* |
| **E-DII** |  |  |  |  |  |  |  |
| *Pre* | 0.93 (0.83, 1.05) | 0 | 1.02 (0.89, 1.17) | 0 |  | 0.99 (0.82, 1.20) | 0 |
| Np/Nc | 2855/2 |  | 2409/2 |  |  | 1516/2 |  |
| *Preg* | 0.94 (0.87, 1.02) | 0 | 0.95 (0.89, 1.02) | 0 |  | 1.07 (0.97, 1.17) | 34 |
| Np/Nc | 5614/6 |  | 8070/7 |  |  | 10435/5 |  |
| *Early* | 0.98 (0.89, 1.08) | 0 | 0.95 (0.89, 1.02) | 0 |  | 1.09 (0.99, 1.19) | 0 |
| Np/Nc | 3868/4 |  | 6495/5 |  |  | 4734/3 |  |
| *Late* | 0.90 (0.82, 0.99)* | 0 | 0.98 (0.87, 1.10) | 0 |  | 1.09 (0.91, 1.30) | 54 |
| Np/Nc | 3624/3 |  | 3077/3 |  |  | 6669/3 |  |
|  |  |  |  |  |  |  |  |
| **DASH** |  |  |  |  |  |  |  |
| *Pre* | 1.01 (0.87, 1.16) | 20 | 0.93 (0.80, 1.07) | 0 |  | 0.94 (0.77, 1.13) | 0 |
| Np/Nc | 2855/2 |  | 2409/2 |  |  | 1516/2 |  |
| *Preg* | 0.98 (0.90, 1.07) | 0 | 1.05 (0.95, 1.15) | 26 |  | 0.94 (0.89, 1.004) | 0 |
| Np/Nc | 5614/6 |  | 8070/7 |  |  | 10435/5 |  |
| *Early* | 0.97 (0.88, 1.07) | 0 | 1.08 (0.96, 1.21) | 33 |  | 0.86 (0.70, 1.06) | 60 |
| Np/Nc | 3868/4 |  | 6495/5 |  |  | 4734/3 |  |
| *Late* | 1.03 (0.93, 1.13) | 0 | 0.98 (0.87, 1.11) | 0 |  | 0.94 (0.87, 1.02) | 0 |
| Np/Nc | 3624/3 |  | 3077/3 |  |  | 6669/3 |  |

Values are adjusted pooled effect estimates [OR (95% CI)] expressed for a 1-SD increment in dietary scores, heterogeneity measure (*I*^2^), and number of participants and studies included (Np/Nc) across different outcomes and conception periods, as labelled. Effect estimates were adjusted for maternal education, ethnicity, pre-pregnancy BMI, maternal height, parity, energy intake (for DASH analysis only), cigarette smoking and alcohol consumption during pregnancy, and (intrinsically adjusted for the outcome) child sex and age at measurement.

E-DII, energy-adjusted Dietary Inflammatory Index; DASH, Dietary Approaches to Stop Hypertension; OWOB, overweight and obesity; *I*^2^, *I*-squared; Pre, pre-pregnancy; Preg, pregnancy; Early, early pregnancy; Late, late pregnancy; Np, number of participants included; Nc, number of cohorts included.

**P*<0.05, ***P*<0.01

**Additional File 1: Table S9** Association between maternal E-DII and DASH scores (per 1-SD increase) and secondary childhood adiposity measures- excluding mothers with pregnancy complications

|  | Early-childhood  (2.8 ± 0.3 year) | |  | Mid-childhood  (6.1 ± 0.6 y) | | | | | |  | Late-childhood  (10.6 ± 1.2 y) | | | | | |
| --- | --- | --- | --- | --- | --- | --- | --- | --- | --- | --- | --- | --- | --- | --- | --- | --- |
|  | SST, mm |  |  | SST, mm |  | FMI, kg/m^2^ |  | FFMI, kg/m^2^ |  |  | SST, mm |  | FMI, kg/m^2^ |  | FFMI, kg/m^2^ |  |
|  | β (95%CI) | *I^2^ (%)* |  | β (95%CI) | *I^2^ (%)* | β (95%CI) | *I^2^ (%)* | β (95%CI) | *I^2^ (%)* |  | β (95%CI) | *I^2^ (%)* | β (95%CI) | *I^2^ (%)* | β (95%CI) | *I^2^ (%)* |
| **E-DII** |  |  |  |  |  |  |  |  |  |  |  |  |  |  |  |  |
| *Pre* | -0.11 (-0.23, 0.02) | 0 |  | -0.05 (-0.30, 0.19) | 36 | 0.001 (-0.04, 0.04) | 0 | -0.03 (-0.08, 0.01) | 0 |  | -0.06 (0.70, 0.55) | - | 0.04 (-0.10, 0.17) | - | -0.02 (-0.10, 0.06) | - |
| Np/Nc | 2706/2 |  |  | 2318/2 |  | 1858/2 |  | 1861/2 |  |  | 956/1 |  | 790/1 |  | 790/1 |  |
| *Preg* | -0.00 (-0.13, 0.13) | 0 |  | 0.11 (-0.10, 0.32) | 13 | 0.03 (-0.03, 0.09) | 28 | -0.06 (-0.16, 0.04) | 70* |  | 0.36 (-0.31, 1.03) | - | 0.03 (-0.04, 0.10) | 0 | -0.06 (-0.09, -0.02)*** | 0 |
| Np/Nc | 2524/3 |  |  | 2878/4 |  | 1894/4 |  | 1897/4 |  |  | 724/1 |  | 5730/2 |  | 5730/2 |  |
| *Early* | 0.08 (-0.10, 0.25) | 0 |  | -0.02 (-0.29, 0.26) | 0 | 0.03 (-0.09, 0.14) | 40 | -0.12 (-0.33, 0.09) | 80** |  | 0.22 (-0.43, 0.87) | - | 0.05 (-0.09, 0.18) | - | -0.04 (-0.12, 0.05) | - |
| Np/Nc | 1603/2 |  |  | 1428/2 |  | 1063/3 |  | 1062/3 |  |  | 773/1 |  | 649/1 |  | 649/1 |  |
| *Late* | -0.03 (-0.16, 0.10) | 0 |  | 0.16 (-0.03, 0.35) | 0 | 0.03 (-0.01, 0.07) | 0 | 0.01 (-0.04, 0.05) | 0 |  | 0.33 (-0.28, 0.93) | - | 0.04 (-0.03, 0.10) | 0 | -0.04 (-0.09, 0.01) | 40 |
| Np/Nc | 2640/2 |  |  | 2865/3 |  | 1810/2 |  | 1813/2 |  |  | 907/1 |  | 5871/2 |  | 5871/2 |  |
|  |  |  |  |  |  |  |  |  |  |  |  |  |  |  |  |  |
| **DASH** |  |  |  |  |  |  |  |  |  |  |  |  |  |  |  |  |
| *Pre* | -0.05 (-0.20, 0.09) | 21 |  | -0.17 (-0.44, 0.09) | 43 | -0.04 (-0.08, 0.004) | 0 | 0.04 (-0.01, 0.09) | 0 |  | -0.35 (-0.96, 0.26) | - | -0.09 (-0.23, 0.04) | - | 0.06 (-0.02, 0.14) | - |
| Np/Nc | 2706/2 |  |  | 2318/2 |  | 1858/2 |  | 1861/2 |  |  | 956/1 |  | 790/1 |  | 790/1 |  |
| *Preg* | -0.08 (-0.21, 0.06) | 0 |  | -0.002 (-0.24, 0.23) | 22 | -0.01 (-0.05, 0.03) | 0 | 0.01 (-0.03, 0.06) | 0 |  | -0.50 (-1.19, 0.19) | - | -0.07 (-0.13, -0.001)* | 0 | 0.02 (-0.01, 0.05) | 0 |
| Np/Nc | 2524/3 |  |  | 2878/4 |  | 1894/4 |  | 1897/4 |  |  | 724/1 |  | 5730/2 |  | 5730/2 |  |
| *Early* | -0.12 (-0.30, 0.07) | 0 |  | 0.09 (-0.46, 0.63) | 71 | -0.08 (-0.16, 0.001) | 0 | 0.03 (-0.03, 0.10) | 0 |  | -0.60 (-1.28, 0.07) | - | -0.14 (-0.28, 0.00) | - | 0.08 (-0.01, 0.17) | - |
| Np/Nc | 1603/2 |  |  | 1428/2 |  | 1063/3 |  | 1062/3 |  |  | 773/1 |  | 649/1 |  | 649/1 |  |
| *Late* | -0.03 (-0.16, 0.10) | 0 |  | -0.01 (-0.21, 0.18) | 0 | 0.001 (-0.04, 0.04) | 0 | -0.00 (-0.05, 0.05) | 0 |  | -0.18 (-0.80, 0.43) | - | -0.05 (-0.11, 0.02) | 0 | 0.02 (-0.01, 0.05) | 0 |
| Np/Nc | 2640/2 |  |  | 2865/3 |  | 1810/2 |  | 1813/2 |  |  | 907/1 |  | 5871/2 |  | 6871/2 |  |

Values are adjusted pooled effect estimates [β (95% CI)] expressed for a 1-SD increment in dietary scores, heterogeneity measure (*I*^2^), and number of participants and studies included (Np/Nc) across different outcomes and conception periods, as labelled. Effect estimates were adjusted for maternal education, ethnicity, pre-pregnancy BMI, maternal height, parity, energy intake (for DASH analysis only), cigarette smoking and alcohol consumption during pregnancy, and child sex and age at measurement.

E-DII, energy-adjusted Dietary Inflammatory Index; DASH, Dietary Approaches to Stop Hypertension; SST, sum of skinfold thickness; FMI, fat mass index; FFMI, fat free mass index; *I*^2^, *I*-squared; Pre, pre-pregnancy; Preg, pregnancy; Early, early pregnancy; Late, late pregnancy; Np, number of participants included; Nc, number of cohorts included.

**P*<0.05, ***P*<0.01

**Additional File 1: Table S10** Association between maternal E-DII and DASH scores (per 1-SD increase) and childhood OWOB- with mutual adjustment of E-DII and DASH

|  | Early-childhood |  | Mid-childhood |  |  | Late-childhood |  |
| --- | --- | --- | --- | --- | --- | --- | --- |
|  | OR (95%CI) | *I^2^ (%)* | OR (95%CI) | *I^2^ (%)* |  | OR (95%CI) | *I^2^ (%)* |
| **E-DII** |  |  |  |  |  |  |  |
| *Pre* | 0.94 (0.82, 1.08) | 0 | 0.95 (0.80, 1.11) | 0 |  | 0.90 (0.72, 1.13) | 0 |
| Np/Nc | 3122/2 |  | 2635/2 |  |  | 1658/2 |  |
| *Preg* | 0.90 (0.82, 0.99)* | 0 | 0.96 (0.86, 1.07) | 27 |  | 1.04 (0.94, 1.16) | 35 |
| Np/Nc | 6111/6 |  | 8717/7 |  |  | 11803/5 |  |
| *Early* | 0.92 (0.82, 1.03) | 0 | 0.97 (0.85, 1.11) | 35 |  | 0.98 (0.79, 1.22) | 51 |
| Np/Nc | 4103/4 |  | 6903/5 |  |  | 5065/3 |  |
| *Late* | 0.90 (0.81, 1.01) | 0 | 0.94 (0.82, 1.08) | 0 |  | 1.02 (0.90, 1.16) | 22 |
| Np/Nc | 4027/3 |  | 3419/3 |  |  | 7779/3 |  |
|  |  |  |  |  |  |  |  |
| **DASH** |  |  |  |  |  |  |  |
| *Pre* | 0.95 (0.75, 1.20) | 54 | 0.90 (0.76, 1.06) | 0 |  | 0.93 (0.70, 1.24) | 28 |
| Np/Nc | 3122/2 |  | 2635/2 |  |  | 1658/2 |  |
| *Preg* | 0.93 (0.84, 1.02) | 0 | 0.999 (0.88, 1.13) | 37 |  | 0.94 (0.85, 1.03) | 27 |
| Np/Nc | 6111/6 |  | 8717/7 |  |  | 11803/5 |  |
| *Early* | 0.94 (0.82, 1.08) | 17 | 1.04 (0.90, 1.19) | 38 |  | 0.83 (0.60, 1.13) | 75* |
| Np/Nc | 4103/4 |  | 6903/5 |  |  | 5065/3 |  |
| *Late* | 0.95 (0.85, 1.07) | 0 | 0.93 (0.81, 1.07) | 0 |  | 0.92 (0.85, 0.99)* | 0 |
| Np/Nc | 4027/3 |  | 3419/3 |  |  | 7779/3 |  |

Values are adjusted pooled effect estimates [OR (95% CI)] expressed for a 1-SD increment in dietary scores, heterogeneity measure (*I*^2^), and number of participants and studies included (Np/Nc) across different outcomes and conception periods, as labelled. Effect estimates were adjusted for maternal education, ethnicity, pre-pregnancy BMI, maternal height, parity, energy intake (for DASH analysis only), cigarette smoking and alcohol consumption during pregnancy, and (intrinsically adjusted for the outcome) child sex and age at measurement.

E-DII, energy-adjusted Dietary Inflammatory Index; DASH, Dietary Approaches to Stop Hypertension; OWOB, overweight and obesity; *I*^2^, *I*-squared; Pre, pre-pregnancy; Preg, pregnancy; Early, early pregnancy; Late, late pregnancy; Np, number of participants included; Nc, number of cohorts included.

**P*<0.05, ***P*<0.01

**Additional File 1: Table S11** Association between maternal E-DII and DASH scores (per 1-SD increase) and secondary childhood adiposity measures- with mutual adjustment of E-DII and DASH

|  | Early-childhood  (2.8 ± 0.3 year) | |  | Mid-childhood  (6.1 ± 0.6 y) | | | | | |  | Late-childhood  (10.6 ± 1.2 y) | | | | | |
| --- | --- | --- | --- | --- | --- | --- | --- | --- | --- | --- | --- | --- | --- | --- | --- | --- |
|  | SST, mm |  |  | SST, mm |  | FMI, kg/m^2^ |  | FFMI, kg/m^2^ |  |  | SST, mm |  | FMI, kg/m^2^ |  | FFMI, kg/m^2^ |  |
|  | β (95%CI) | *I^2^ (%)* |  | β (95%CI) | *I^2^ (%)* | β (95%CI) | *I^2^ (%)* | β (95%CI) | *I^2^ (%)* |  | β (95%CI) | *I^2^ (%)* | β (95%CI) | *I^2^ (%)* | β (95%CI) | *I^2^ (%)* |
| **E-DII** |  |  |  |  |  |  |  |  |  |  |  |  |  |  |  |  |
| *Pre* | -0.18 (-0.35, -0.02)* | 17 |  | -0.19 (-0.41, 0.03) | 0 | -0.04 (-0.08, 0.01) | 0 | -0.03 (-0.08, 0.03) | 0 |  | -0.54 (-1.28, 0.21) | - | -0.07 (-0.23, 0.09) | - | -0.002 (-0.09, 0.09) | - |
| Np/Nc | 2959/2 |  |  | 2536/2 |  | 2034/2 |  | 2038/2 |  |  | 1025/1 |  | 848/1 |  | 848/1 |  |
| *Preg* | -0.05 (-0.20, 0.11) | 0 |  | 0.12 (-0.19, 0.42) | 30 | 0.02 (-0.06, 0.10) | 32 | -0.06 (-0.16, 0.05) | 54 |  | -0.01 (-0.84, 0.81) | - | 0.01 (-0.06, 0.08) | 0 | -0.05 (-0.08, -0.02)** | 0 |
| Np/Nc | 2749/3 |  |  | 3184/4 |  | 2059/4 |  | 2063/4 |  |  | 780/1 |  | 6739/2 |  | 6739/2 |  |
| *Early* | 0.05 (-0.16, 0.26) | 0 |  | -0.02 (-0.35, 0.31) | 0 | -0.03 (-0.21, 0.14) | 58 | -0.13 (-0.34, 0.09) | 71* |  | -0.26 (-1.02, 0.50) | - | -0.05 (-0.22, 0.11) | - | -0.04 (-0.14, 0.06) | - |
| Np/Nc | 1716/2 |  |  | 1513/2 |  | 1120/3 |  | 1119/3 |  |  | 836/1 |  | 702/1 |  | 702/1 |  |
| *Late* | -0.07 (-0.22, 0.09) | 0 |  | 0.18 (-0.17, 0.54) | 38 | 0.04 (-0.01, 0.09) | 0 | -0.01 (-0.06, 0.05) | 0 |  | 0.08 (-0.65, 0.81) | - | 0.03 (-0.04, 0.10) | 0 | -0.04 (-0.09, 0.02) | 32 |
| Np/Nc | 2880/2 |  |  | 3182/3 |  | 1980/2 |  | 1984/2 |  |  | 969/1 |  | 6885/2 |  | 6885/2 |  |
|  |  |  |  |  |  |  |  |  |  |  |  |  |  |  |  |  |
| **DASH** |  |  |  |  |  |  |  |  |  |  |  |  |  |  |  |  |
| *Pre* | -0.14 (-0.37, 0.09) | 55 |  | -0.29 (-0.52, -0.07)* | 0 | -0.06 (-0.11, -0.01)* | 0 | 0.02 (-0.04, 0.07) | 0 |  | -0.60 (-1.33, 0.14) | - | -0.14 (-0.30, 0.02) | - | 0.05 (-0.05, 0.14) | - |
| Np/Nc | 2959/2 |  |  | 2536/2 |  | 2034/2 |  | 2038/2 |  |  | 1025/1 |  | 848/1 |  | 848/1 |  |
| *Preg* | -0.08 (-0.24, 0.08) | 0 |  | 0.05 (-0.30, 0.40) | 43 | 0.001 (-0.05, 0.05) | 0 | 0.002 (-0.06, 0.06) | 0 |  | -0.55 (-1.38, 0.28) | - | -0.08 (-0.15, -0.01)* | 0 | 0.01 (-0.03, 0.04) | 0 |
| Np/Nc | 2749/3 |  |  | 3184/4 |  | 2059/4 |  | 2063/4 |  |  | 780/1 |  | 6739/2 |  | 6739/2 |  |
| *Early* | -0.08 (-0.29, 0.14) | 0 |  | 0.08 (-0.44, 0.61) | 57 | -0.08 (-0.18, 0.02) | 5 | 0.02 (-0.05, 0.09) | 0 |  | -0.72 (-1.48, 0.05) | - | -0.15 (-0.31, 0.01) | - | 0.04 (-0.06, 0.14) | - |
| Np/Nc | 1716/2 |  |  | 1513/2 |  | 1120/3 |  | 1119/3 |  |  | 836/1 |  | 702/1 |  | 702/1 |  |
| *Late* | -0.05 (-0.20, 0.10) | 0 |  | 0.06 (-0.29, 0.41) | 38 | 0.02 (-0.03, 0.06) | 0 | -0.004 (-0.06, 0.05) | 0 |  | -0.23 (-0.96, 0.50) | - | -0.06 (-0.13, 0.01) | 0 | 0.01 (-0.02, 0.04) | 0 |
| Np/Nc | 2880/2 |  |  | 3182/3 |  | 1980/2 |  | 1984/2 |  |  | 969/1 |  | 6885/2 |  | 6885/2 |  |

Values are adjusted pooled effect estimates [β (95% CI)] expressed for a 1-SD increment in dietary scores, heterogeneity measure (*I*^2^), and number of participants and studies included (Np/Nc) across different outcomes and conception periods, as labelled. Effect estimates were adjusted for maternal education, ethnicity, pre-pregnancy BMI, maternal height, parity, energy intake (for DASH analysis only), cigarette smoking and alcohol consumption during pregnancy, and child sex and age at measurement.

E-DII, energy-adjusted Dietary Inflammatory Index; DASH, Dietary Approaches to Stop Hypertension; SST, sum of skinfold thickness; FMI, fat mass index; FFMI, fat free mass index; *I*^2^, *I*-squared; Pre, pre-pregnancy; Preg, pregnancy; Early, early pregnancy; Late, late pregnancy; Np, number of participants included; Nc, number of cohorts included.

**P*<0.05, ***P*<0.01

**Additional File 1: Table S12** Association between maternal E-DII and DASH scores (per 1-SD increase) and childhood obesity (BMI z-score >95^th^ percentile)

|  | Early-childhood |  | Mid-childhood |  |  | Late-childhood |  |
| --- | --- | --- | --- | --- | --- | --- | --- |
|  | OR (95%CI) | *I^2^ (%)* | OR (95%CI) | *I^2^ (%)* |  | OR (95%CI) | *I^2^ (%)* |
| **E-DII** |  |  |  |  |  |  |  |
| *Pre* | 0.95 (0.79, 1.13) | 0 | 0.96 (0.77, 1.20) | 0 |  | 1.26 (0.65, 2.46) | 71 |
| Np/Nc | 3122/2 |  | 2635/2 |  |  | 1658/2 |  |
| *Preg* | 0.95 (0.84, 1.07) | 0 | 0.95 (0.81, 1.11) | 36 |  | 1.15 (0.98, 1.36) | 45 |
| Np/Nc | 6094/6 |  | 8709/7 |  |  | 11803/5 |  |
| *Early* | 0.97 (0.84, 1.13) | 0 | 0.97 (0.82, 1.14) | 32 |  | 1.12 (0.97, 1.29) | 0 |
| Np/Nc | 4101/4 |  | 6895/5 |  |  | 5065/3 |  |
| *Late* | 0.99 (0.85, 1.15) | 0 | 0.997 (0.72, 1.39) | 57 |  | 1.25 (0.86, 1.83) | 73 |
| Np/Nc | 4012/3 |  | 3419/3 |  |  | 7779/3 |  |
|  |  |  |  |  |  |  |  |
| **DASH** |  |  |  |  |  |  |  |
| *Pre* | 0.95 (0.80, 1.15) | 0 | 0.89 (0.71, 1.13) | 0 |  | 0.94 (0.70, 1.26) | 0 |
| Np/Nc | 3122/2 |  | 2635/2 |  |  | 1658/2 |  |
| *Preg* | 0.90 (0.77, 1.04) | 16 | 1.05 (0.82, 1.33) | 67** |  | 0.88 (0.80, 0.96)** | 0 |
| Np/Nc | 6094/6 |  | 8709/7 |  |  | 11803/5 |  |
| *Early* | 0.88 (0.71, 1.10) | 34 | 1.09 (0.78, 1.53) | 79** |  | 0.87 (0.75, 1.02) | 0 |
| Np/Nc | 4101/4 |  | 6895/5 |  |  | 5065/3 |  |
| *Late* | 0.90 (0.77, 1.05) | 0 | 0.94 (0.77, 1.15) | 0 |  | 0.86 (0.64, 1.17) | 59 |
| Np/Nc | 4012/3 |  | 3419/3 |  |  | 7779/3 |  |

Values are adjusted pooled effect estimates [OR (95% CI)] expressed for a 1-SD increment in dietary scores, heterogeneity measure (*I*^2^), and number of participants and studies included (Np/Nc) across different outcomes and conception periods, as labelled. Effect estimates were adjusted for maternal education, ethnicity, pre-pregnancy BMI, maternal height, parity, energy intake (for DASH analysis only), cigarette smoking and alcohol consumption during pregnancy, and (intrinsically adjusted for the outcome) child sex and age at measurement.

E-DII, energy-adjusted Dietary Inflammatory Index; DASH, Dietary Approaches to Stop Hypertension; OWOB, overweight and obesity; *I*^2^, *I*-squared; Pre, pre-pregnancy; Preg, pregnancy; Early, early pregnancy; Late, late pregnancy; Np, number of participants included; Nc, number of cohorts included.

**P*<0.05, ***P*<0.01

**Additional File 1: Table S13** Association between maternal E-DII and DASH scores (per 1-SD increase) and childhood OWOB- with further adjustment of birthweight

|  | Early-childhood |  | Mid-childhood |  |  | Late-childhood |  |
| --- | --- | --- | --- | --- | --- | --- | --- |
|  | OR (95%CI) | *I^2^ (%)* | OR (95%CI) | *I^2^ (%)* |  | OR (95%CI) | *I^2^ (%)* |
| **E-DII** |  |  |  |  |  |  |  |
| *Pre* | 0.95 (0.85, 1.06) | 0 | 1.02 (0.89, 1.16) | 0 |  | 0.96 (0.80, 1.15) | 0 |
| Np/Nc | 3110/2 |  | 2628/2 |  |  | 1653/2 |  |
| *Preg* | 0.96 (0.89, 1.03) | 0 | 0.98 (0.92, 1.04) | 0 |  | 1.07 (0.99, 1.15) | 23 |
| Np/Nc | 6084/6 |  | 8697/7 |  |  | 11719/5 |  |
| *Early* | 0.99 (0.90, 1.09) | 0 | 0.98 (0.91, 1.05) | 4 |  | 1.10 (1.004, 1.20)* | 0 |
| Np/Nc | 4083/4 |  | 6888/5 |  |  | 5056/3 |  |
| *Late* | 0.92 (0.84, 1.01) | 0 | 0.98 (0.87, 1.09) | 0 |  | 1.07 (0.94, 1.21) | 35 |
| Np/Nc | 4008/3 |  | 3407/3 |  |  | 7699/3 |  |
|  |  |  |  |  |  |  |  |
| **DASH** |  |  |  |  |  |  |  |
| *Pre* | 0.97 (0.79, 1.20) | 60 | 0.92 (0.80, 1.06) | 0 |  | 0.96 (0.80, 1.15) | 0 |
| Np/Nc | 3110/2 |  | 2628/2 |  |  | 1653/2 |  |
| *Preg* | 0.97 (0.89, 1.05) | 0 | 1.02 (0.95, 1.10) | 3 |  | 0.92 (0.87, 0.97)** | 0 |
| Np/Nc | 6084/6 |  | 8697/7 |  |  | 11719/5 |  |
| *Early* | 0.97 (0.88, 1.07) | 0 | 1.04 (0.95, 1.13) | 13 |  | 0.85 (0.70, 1.04) | 57 |
| Np/Nc | 4083/4 |  | 6888/5 |  |  | 5056/3 |  |
| *Late* | 0.995 (0.90, 1.10) | 0 | 0.95 (0.85, 1.07) | 0 |  | 0.91 (0.85, 0.98)** | 0 |
| Np/Nc | 4008/3 |  | 3407/3 |  |  | 7699/3 |  |

Values are adjusted pooled effect estimates [OR (95% CI)] expressed for a 1-SD increment in dietary scores, heterogeneity measure (*I*^2^), and number of participants and studies included (Np/Nc) across different outcomes and conception periods, as labelled. Effect estimates were adjusted for maternal education, ethnicity, pre-pregnancy BMI, maternal height, parity, energy intake (for DASH analysis only), cigarette smoking and alcohol consumption during pregnancy, and (intrinsically adjusted for the outcome) child sex and age at measurement. Results for this table were further adjusted for birthweight.

E-DII, energy-adjusted Dietary Inflammatory Index; DASH, Dietary Approaches to Stop Hypertension; OWOB, overweight and obesity; *I*^2^, *I*-squared; Pre, pre-pregnancy; Preg, pregnancy; Early, early pregnancy; Late, late pregnancy; Np, number of participants included; Nc, number of cohorts included.

**P*<0.05, ***P*<0.01

**Additional File 1: Table S14** Association between maternal E-DII and DASH scores (per 1-SD increase) and secondary childhood adiposity measures- with further adjustment of birthweight

|  | Early-childhood  (2.8 ± 0.3 year) | |  | Mid-childhood  (6.1 ± 0.6 y) | | | | | |  | Late-childhood  (10.6 ± 1.2 y) | | | | | |
| --- | --- | --- | --- | --- | --- | --- | --- | --- | --- | --- | --- | --- | --- | --- | --- | --- |
|  | SST, mm |  |  | SST, mm |  | FMI, kg/m^2^ |  | FFMI, kg/m^2^ |  |  | SST, mm |  | FMI, kg/m^2^ |  | FFMI, kg/m^2^ |  |
|  | β (95%CI) | *I^2^ (%)* |  | β (95%CI) | *I^2^ (%)* | β (95%CI) | *I^2^ (%)* | β (95%CI) | *I^2^ (%)* |  | β (95%CI) | *I^2^ (%)* | β (95%CI) | *I^2^ (%)* | β (95%CI) | *I^2^ (%)* |
| **E-DII** |  |  |  |  |  |  |  |  |  |  |  |  |  |  |  |  |
| *Pre* | -0.10 (-0.22, 0.02) | 0 |  | -0.03 (-0.21, 0.15) | 0 | -0.003 (-0.04, 0.04) | 0 | -0.04 (-0.08, 0.01) | 0 |  | -0.19 (-0.77, 0.40) | - | 0.02 (-0.10, 0.15) | - | -0.03 (-0.10, 0.04) | - |
| Np/Nc | 2948/2 |  |  | 2529/2 |  | 2029/2 |  | 2033/2 |  |  | 1020/1 |  | 844/1 |  | 844/1 |  |
| *Preg* | -0.01 (-0.14, 0.13) | 10 |  | 0.09 (-0.16, 0.33) | 33 | 0.03 (-0.02, 0.08) | 15 | -0.06 (-0.16, 0.03) | 65* |  | 0.37 (-0.27, 1.02) | - | 0.04 (-0.03, 0.10) | 0 | -0.05 (-0.08, -0.02)*** | 0 |
| Np/Nc | 2740/3 |  |  | 3177/4 |  | 2055/4 |  | 2059/4 |  |  | 777/1 |  | 6660/2 |  | 6660/2 |  |
| *Early* | 0.07 (-0.10, 0.24) | 0 |  | -0.02 (-0.28, 0.25) | 1 | 0.01 (-0.10, 0.12) | 35 | -0.13 (-0.32, 0.06) | 76* |  | 0.16 (-0.46, 0.79) | - | 0.03 (-0.10, 0.16) | - | -0.05 (-0.13, 0.03) | - |
| Np/Nc | 1706/2 |  |  | 1507/2 |  | 1115/3 |  | 1114/3 |  |  | 832/1 |  | 698/1 |  | 698/1 |  |
| *Late* | -0.04 (-0.16, 0.08) | 0 |  | 0.16 (-0.06, 0.38) | 16 | 0.03 (-0.01, 0.07) | 0 | -0.002 (-0.05, 0.04) | 0 |  | 0.28 (-0.31, 0.87) | - | 0.04 (-0.02, 0.10) | 0 | -0.04 (-0.08, -0.01)* | 18 |
| Np/Nc | 2870/2 |  |  | 3174/3 |  | 1976/2 |  | 1980/2 |  |  | 965/1 |  | 6806/2 |  | 6806/2 |  |
|  |  |  |  |  |  |  |  |  |  |  |  |  |  |  |  |  |
| **DASH** |  |  |  |  |  |  |  |  |  |  |  |  |  |  |  |  |
| *Pre* | -0.05 (-0.17, 0.08) | 0 |  | -0.19 (-0.37, -0.003)* | 0 | -0.04 (-0.08, 0.002) | 0 | 0.03 (-0.02, 0.07) | 0 |  | -0.31 (-0.90, 0.28) | - | -0.10 (-0.23, 0.03) | - | 0.04 (-0.03, 0.11) | - |
| Np/Nc | 2948/2 |  |  | 2529/2 |  | 2029/2 |  | 2033/2 |  |  | 1020/1 |  | 844/1 |  | 844/1 |  |
| *Preg* | -0.06 (-0.19, 0.06) | 0 |  | -0.02 (-0.30, 0.26) | 42 | -0.02 (-0.06, 0.02) | 0 | 0.01 (-0.04, 0.06) | 9 |  | -0.51 (-1.17, 0.15) | - | -0.09 (-0.15, -0.02)** | 0 | 0.02 (-0.01, 0.05) | 0 |
| Np/Nc | 2740/3 |  |  | 3177/4 |  | 2055/4 |  | 2059/4 |  |  | 777/1 |  | 6660/2 |  | 6660/2 |  |
| *Early* | -0.11 (-0.29, 0.07) | 0 |  | 0.09 (-0.46, 0.64) | 72 | -0.07 (-0.15, 0.01) | 0 | 0.05 (-0.05, 0.15) | 26 |  | -0.59 (-1.23, 0.05) | - | -0.12 (-0.25, 0.01) | - | 0.06 (-0.02, 0.14) | - |
| Np/Nc | 1706/2 |  |  | 1507/2 |  | 1115/3 |  | 1114/3 |  |  | 832/1 |  | 698/1 |  | 698/1 |  |
| *Late* | -0.02 (-0.15, 0.10) | 0 |  | -0.07 (-0.25, 0.12) | 0 | -0.01 (-0.05, 0.03) | 0 | -0.01 (-0.05, 0.04) | 0 |  | -0.26 (-0.86, 0.34) | - | -0.07 (-0.13, -0.01)* | 0 | 0.02 (-0.01, 0.05) | 0 |
| Np/Nc | 2870/2 |  |  | 3174/3 |  | 1976/2 |  | 1980/2 |  |  | 965/1 |  | 6806/2 |  | 6806/2 |  |

Values are adjusted pooled effect estimates [β (95% CI)] expressed for a 1-SD increment in dietary scores, heterogeneity measure (*I*^2^), and number of participants and studies included (Np/Nc) across different outcomes and conception periods, as labelled. Effect estimates were adjusted for maternal education, ethnicity, pre-pregnancy BMI, maternal height, parity, energy intake (for DASH analysis only), cigarette smoking and alcohol consumption during pregnancy, and child sex and age at measurement. Results for this table were further adjusted for birthweight.

E-DII, energy-adjusted Dietary Inflammatory Index; DASH, Dietary Approaches to Stop Hypertension; SST, sum of skinfold thickness; FMI, fat mass index; FFMI, fat free mass index; *I*^2^, *I*-squared; Pre, pre-pregnancy; Preg, pregnancy; Early, early pregnancy; Late, late pregnancy; Np, number of participants included; Nc, number of cohorts included.

**P*<0.05, ***P*<0.01

**Additional File 1: Table S15** Association between maternal E-DII and DASH scores (per 1-SD increase) and childhood OWOB- with further adjustment of gestational age

|  | Early-childhood |  | Mid-childhood |  |  | Late-childhood |  |
| --- | --- | --- | --- | --- | --- | --- | --- |
|  | OR (95%CI) | *I^2^ (%)* | OR (95%CI) | *I^2^ (%)* |  | OR (95%CI) | *I^2^ (%)* |
| **E-DII** |  |  |  |  |  |  |  |
| *Pre* | 0.94 (0.84, 1.05) | 0 | 1.01 (0.88, 1.15) | 0 |  | 0.96 (0.81, 1.15) | 0 |
| Np/Nc | 3122/2 |  | 2635/2 |  |  | 1658/2 |  |
| *Preg* | 0.95 (0.88, 1.02) | 0 | 0.97 (0.91, 1.03) | 0 |  | 1.07 (0.98, 1.17) | 36 |
| Np/Nc | 6111/6 |  | 8674/7 |  |  | 11783/5 |  |
| *Early* | 0.98 (0.89, 1.08) | 0 | 0.97 (0.91, 1.04) | 0 |  | 1.09 (0.997, 1.19) | 0 |
| Np/Nc | 4103/4 |  | 6860/5 |  |  | 5045/3 |  |
| *Late* | 0.91 (0.83, 0.998)* | 0 | 0.97 (0.85, 1.11) | 24 |  | 1.08 (0.93, 1.26) | 48 |
| Np/Nc | 4027/3 |  | 3419/3 |  |  | 7779/3 |  |
|  |  |  |  |  |  |  |  |
| **DASH** |  |  |  |  |  |  |  |
| *Pre* | 0.99 (0.80, 1.22) | 62 | 0.93 (0.81, 1.06) | 0 |  | 0.96 (0.80, 1.15) | 0 |
| Np/Nc | 3122/2 |  | 2635/2 |  |  | 1658/2 |  |
| *Preg* | 0.98 (0.91, 1.06) | 0 | 1.03 (0.96, 1.11) | 4 |  | 0.93 (0.87, 0.98)** | 0 |
| Np/Nc | 6111/6 |  | 8674/7 |  |  | 11783/5 |  |
| *Early* | 0.99 (0.90, 1.09) | 0 | 1.05 (0.97, 1.15) | 8 |  | 0.89 (0.74, 1.06) | 47 |
| Np/Nc | 4103/4 |  | 6860/5 |  |  | 5045/3 |  |
| *Late* | 1.01 (0.92, 1.11) | 0 | 0.96 (0.85, 1.08) | 0 |  | 0.91 (0.85, 0.98)* | 0 |
| Np/Nc | 4027/3 |  | 3419/3 |  |  | 7779/3 |  |

Values are adjusted pooled effect estimates [OR (95% CI)] expressed for a 1-SD increment in dietary scores, heterogeneity measure (*I*^2^), and number of participants and studies included (Np/Nc) across different outcomes and conception periods, as labelled. Effect estimates were adjusted for maternal education, ethnicity, pre-pregnancy BMI, maternal height, parity, energy intake (for DASH analysis only), cigarette smoking and alcohol consumption during pregnancy, and (intrinsically adjusted for the outcome) child sex and age at measurement. Results for this table were further adjusted for gestational age.

E-DII, energy-adjusted Dietary Inflammatory Index; DASH, Dietary Approaches to Stop Hypertension; OWOB, overweight and obesity; *I*^2^, *I*-squared; Pre, pre-pregnancy; Preg, pregnancy; Early, early pregnancy; Late, late pregnancy; Np, number of participants included; Nc, number of cohorts included.

**P*<0.05, ***P*<0.01

**Additional File 1: Table S16** Association between maternal E-DII and DASH scores (per 1-SD increase) and secondary childhood adiposity measures- with further adjustment of gestational age

|  | Early-childhood  (2.8 ± 0.3 year) | |  | Mid-childhood  (6.1 ± 0.6 y) | | | | | |  | Late-childhood  (10.6 ± 1.2 y) | | | | | |
| --- | --- | --- | --- | --- | --- | --- | --- | --- | --- | --- | --- | --- | --- | --- | --- | --- |
|  | SST, mm |  |  | SST, mm |  | FMI, kg/m^2^ |  | FFMI, kg/m^2^ |  |  | SST, mm |  | FMI, kg/m^2^ |  | FFMI, kg/m^2^ |  |
|  | β (95%CI) | *I^2^ (%)* |  | β (95%CI) | *I^2^ (%)* | β (95%CI) | *I^2^ (%)* | β (95%CI) | *I^2^ (%)* |  | β (95%CI) | *I^2^ (%)* | β (95%CI) | *I^2^ (%)* | β (95%CI) | *I^2^ (%)* |
| **E-DII** |  |  |  |  |  |  |  |  |  |  |  |  |  |  |  |  |
| *Pre* | -0.11 (-0.23, 0.01) | 0 |  | -0.04 (-0.22, 0.14) | 0 | -0.01 (-0.04, 0.03) | 0 | -0.04 (-0.08, 0.003) | 0 |  | -0.17 (-0.76, 0.42) | - | 0.03 (-0.10, 0.16) | - | -0.03 (-0.10, 0.05) | - |
| Np/Nc | 2959/2 |  |  | 2536/2 |  | 2034/2 |  | 2038/2 |  |  | 1025/1 |  | 848/1 |  | 848/1 |  |
| *Preg* | -0.01 (-0.14, 0.12) | 6 |  | 0.09 (-0.14, 0.33) | 28 | 0.03 (-0.03, 0.08) | 23 | -0.07 (-0.17, 0.03) | 69* |  | 0.42 (-0.23, 1.07) | - | 0.03 (-0.03, 0.09) | 0 | -0.06 (-0.08, -0.03)*** | 0 |
| Np/Nc | 2749/3 |  |  | 3184/4 |  | 2058/4 |  | 2062/4 |  |  | 780/1 |  | 6739/2 |  | 6739/2 |  |
| *Early* | 0.07 (-0.11, 0.24) | 0 |  | -0.02 (-0.28, 0.25) | 0 | 0.02 (-0.10, 0.13) | 38 | -0.13 (-0.33, 0.07) | 79** |  | 0.20 (-0.43, 0.82) | - | 0.04 (-0.09, 0.17) | - | -0.04 (-0.12, 0.04) | - |
| Np/Nc | 1716/2 |  |  | 1513/2 |  | 1119/3 |  | 1118/3 |  |  | 836/1 |  | 702/1 |  | 702/1 |  |
| *Late* | -0.04 (-0.16, 0.08) | 0 |  | 0.16 (-0.06, 0.37) | 14 | 0.03 (-0.01, 0.07) | 0 | -0.00 (-0.04, 0.04) | 0 |  | 0.31 (-0.28, 0.90) | - | 0.04 (-0.03, 0.11) | 15 | -0.04 (-0.09, 0.01) | 51 |
| Np/Nc | 2880/2 |  |  | 3182/3 |  | 1980/2 |  | 1984/2 |  |  | 969/1 |  | 6885/2 |  | 6885/2 |  |
|  |  |  |  |  |  |  |  |  |  |  |  |  |  |  |  |  |
| **DASH** |  |  |  |  |  |  |  |  |  |  |  |  |  |  |  |  |
| *Pre* | -0.03 (-0.16, 0.09) | 0 |  | -0.18 (-0.36, 0.01) | 0 | -0.04 (-0.08, 0.004) | 0 | 0.03 (-0.01, 0.08) | 0 |  | -0.29 (-0.88, 0.31) | - | -0.10 (-0.23, 0.03) | - | 0.05 (-0.03, 0.12) | - |
| Np/Nc | 2959/2 |  |  | 2536/2 |  | 2034/2 |  | 2038/2 |  |  | 1025/1 |  | 848/1 |  | 848/1 |  |
| *Preg* | -0.06 (-0.19, 0.07) | 0 |  | -0.02 (-0.30, 0.27) | 42 | -0.02 (-0.05, 0.02) | 0 | 0.01 (-0.03, 0.06) | 0 |  | -0.55 (-1.21, 0.12) | - | -0.09 (-0.15, -0.03)** | 0 | 0.03 (-0.002, 0.06) | 0 |
| Np/Nc | 2749/3 |  |  | 3184/4 |  | 2058/4 |  | 2062/4 |  |  | 780/1 |  | 6739/2 |  | 6739/2 |  |
| *Early* | -0.10 (-0.28, 0.08) | 0 |  | 0.10 (-0.41, 0.61) | 68 | -0.07 (-0.15, 0.01) | 0 | 0.04 (-0.03, 0.11) | 5 |  | -0.58 (-1.22, 0.06) | - | -0.12 (-0.26, 0.01) | - | 0.06 (-0.02, 0.14) | - |
| Np/Nc | 1716/2 |  |  | 1513/2 |  | 1119/3 |  | 1118/3 |  |  | 836/1 |  | 702/1 |  | 702/1 |  |
| *Late* | -0.01 (-0.14, 0.11) | 0 |  | -0.05 (-0.25, 0.14) | 5 | -0.01 (-0.05, 0.03) | 0 | -0.001 (-0.05, 0.04) | 0 |  | -0.28 (-0.88, 0.32) | - | -0.07 (-0.13, -0.01)* | 0 | 0.03 (-0.002, 0.05) | 0 |
| Np/Nc | 2880/2 |  |  | 3182/3 |  | 1980/2 |  | 1984/2 |  |  | 969/1 |  | 6885/2 |  | 6885/2 |  |

Values are adjusted pooled effect estimates [β (95% CI)] expressed for a 1-SD increment in dietary scores, heterogeneity measure (*I*^2^), and number of participants and studies included (Np/Nc) across different outcomes and conception periods, as labelled. Effect estimates were adjusted for maternal education, ethnicity, pre-pregnancy BMI, maternal height, parity, energy intake (for DASH analysis only), cigarette smoking and alcohol consumption during pregnancy, and child sex and age at measurement. Results for this table were further adjusted for gestational age.

E-DII, energy-adjusted Dietary Inflammatory Index; DASH, Dietary Approaches to Stop Hypertension; SST, sum of skinfold thickness; FMI, fat mass index; FFMI, fat free mass index; *I*^2^, *I*-squared; Pre, pre-pregnancy; Preg, pregnancy; Early, early pregnancy; Late, late pregnancy; Np, number of participants included; Nc, number of cohorts included.

**P*<0.05, ***P*<0.01

**Additional File 1: Table S17** Association between maternal E-DII (per 1-SD increase) and late-childhood OWOB and adiposity measures- with and without further adjustment for child E-DII score in cohorts with child E-DII data

|  | Late childhood OWOB | |  | Late-childhood adiposity | | | | | |
| --- | --- | --- | --- | --- | --- | --- | --- | --- | --- |
|  |  |  |  | SST, mm |  | FMI, kg/m^2^ |  | FFMI, kg/m^2^ |  |
|  | OR (95%CI) | *I^2^ (%)* |  | β (95%CI) | *I^2^ (%)* | β (95%CI) | *I^2^ (%)* | β (95%CI) | *I^2^ (%)* |
| **E-DII** |  |  |  |  |  |  |  |  |  |
| *Pre_unadj_* | 0.96 (0.78, 1.18) | - |  | -0.18 (-0.77, 0.42) | - | 0.03 (-0.10, 0.16) | - | -0.03 (-0.10, 0.05) | - |
| *Pre_adj_* | 0.95 (0.77, 1.18) | - |  | -0.23 (-0.84, 0.38) | - | 0.02 (-0.12, 0.15) | - | -0.02 (-0.10, 0.05) | - |
| Np/Nc | 1015/1 |  |  | 1000/1 |  | 833/1 |  | 833/1 |  |
| *Preg_unadj_* | 1.05 (0.98, 1.12) | 0 |  | 0.35 (-0.30, 1.00) | - | 0.04 (-0.03, 0.10) | 0 | -0.05 (-0.08, -0.02)** | 0 |
| *Preg_adj_* | 1.05 (0.98, 1.13) | 0 |  | 0.32 (-0.36, 1.00) | - | 0.01 (-0.06, 0.07) | 0 | -0.04 (-0.07, -0.01)* | 0 |
| Np/Nc | 9460/4 |  |  | 767/1 |  | 6060/2 |  | 6060/2 |  |
| *Early_unadj_* | 1.08 (0.97, 1.21) | 0 |  | 0.15 (-0.49, 0.78) | - | 0.04 (-0.09, 0.17) | - | -0.03 (-0.12, 0.05) | - |
| *Early_adj_* | 1.12 (0.999, 1.25) | 0 |  | 0.10 (-0.55, 0.75) | - | 0.03 (-0.11, 0.16) |  | -0.03 (-0.12, 0.05) |  |
| Np/Nc | 4011/3 |  |  | 816/1 |  | 689/1 |  | 689/1 |  |
| *Late_unadj_* | 1.03 (0.96, 1.11) | 0 |  | 0.27 (-0.32, 0.87) | - | 0.04 (-0.02, 0.11) | 0 | -0.04 (-0.09, 0.01) | 41 |
| *Late_adj_* | 1.02 (0.94, 1.11) | 0 |  | 0.24 (-0.38, 0.85) | - | 0.02 (-0.07, 0.10) | 26 | -0.04 (-0.07, -0.003)* | 0 |
| Np/Nc | 6464/2 |  |  | 951/1 |  | 6204/2 |  | 6204/2 |  |

Values are adjusted pooled effect estimates [OR (95% CI) or β (95% CI)] expressed for a 1-SD increment in dietary scores, heterogeneity measure (*I*^2^), and number of participants and studies included (Np/Nc) across different outcomes and conception periods, as labelled. Effect estimates were adjusted for maternal education, ethnicity, pre-pregnancy BMI, maternal height, parity, energy intake (for DASH analysis only), cigarette smoking and alcohol consumption during pregnancy, and child sex and age at measurement. Row signified by ‘unadj’ subscripts did not include, whereas those with ‘adj’ subscripts included, E-DII in the model.

E-DII, energy-adjusted Dietary Inflammatory Index; DASH, Dietary Approaches to Stop Hypertension; unadj, unadjusted (for E-DII); adj, adjusted (for E-DII). OWOB, overweight and obesity; SST, sum of skinfold thickness; FMI, fat mass index; FFMI, fat free mass index; *I*^2^, *I*-squared; Pre, pre-pregnancy; Preg, pregnancy; Early, early pregnancy; Late, late pregnancy; Np, number of participants included; Nc, number of cohorts included.

**P*<0.05, ***P*<0.01

**Additional File 1: Table S18** Pooled *P*-values for sex-interaction between maternal E-DII and DASH score and offspring adiposity outcomes

|  | Primary outcome | | |  | Early-childhood |  | Mid-childhood | | |  | Late-childhood | | |
| --- | --- | --- | --- | --- | --- | --- | --- | --- | --- | --- | --- | --- | --- |
|  | Early-childhood OWOB | Mid- childhood OWOB | Late-childhood OWOB |  | SST, kg/m^2^ |  | SST, kg/m^2^ | FMI, kg/m^2^ | FFMI, kg/m^2^ |  | SST, kg/m^2^ | FMI, kg/m^2^ | FFMI, kg/m^2^ |
| **E-DII** |  |  |  |  |  |  |  |  |  |  |  |  |  |
| Pre | 0.42 | 0.67 | 0.47 |  | 0.42 |  | 0.46 | 0.12 | 0.68 |  | 0.23 | 0.66 | 0.28 |
| Preg | 0.80 | 0.70 | 0.25 |  | 0.75 |  | 0.97 | 0.031 | 0.62 |  | 0.51 | 0.80 | 0.002 |
| Early | 0.40 | 0.48 | 0.71 |  | 0.69 |  | 0.16 | 0.54 | 0.95 |  | 0.16 | 0.26 | 0.47 |
| Late | 0.53 | 0.84 | 0.099 |  | 0.99 |  | 0.60 | 0.008 | 0.98 |  | 0.70 | 0.45 | 0.002 |
|  |  |  |  |  |  |  |  |  |  |  |  |  |  |
| **DASH** |  |  |  |  |  |  |  |  |  |  |  |  |  |
| Pre | 0.089 | 0.51 | 0.80 |  | 0.44 |  | 0.37 | 0.009 | 0.095 |  | 0.91 | 0.46 | 0.51 |
| Preg | 0.67 | 0.76 | 0.59 |  | 0.22 |  | 0.24 | 0.018 | 0.33 |  | 0.90 | 0.20 | 0.013 |
| Early | 0.57 | 0.89 | 0.83 |  | 0.90 |  | 0.91 | 0.60 | 0.48 |  | 0.73 | 0.36 | 0.59 |
| Late | 0.80 | 0.51 | 0.66 |  | 0.20 |  | 0.19 | 0.095 | 0.11 |  | 0.96 | 0.21 | 0.014 |

E-DII, energy-adjusted Dietary Inflammatory Index; DASH, Dietary Approaches to Stop Hypertension; OWOB, overweight and obesity; SST, sum of skinfold thickness; FMI, fat mass index; FFMI, fat free mass index; Pre, pre-pregnancy; Preg, pregnancy; Early, early pregnancy; Late, late pregnancy

**Additional File 1: Table S19** Stratified estimates for other sex-interactions between maternal E-DII and DASH scores and offspring adiposity outcomes (all *P*-interactions <0.10)

| Group | Period | Exposure | Outcome | OR (95% CI) |
| --- | --- | --- | --- | --- |
| Male | Late-pregnancy | E-DII | Late-childhood overweight & obesity | 1.09 (0.85, 1.40) |
| Female | Late-pregnancy | E-DII | Late-childhood  overweight & obesity | 1.07 (0.97, 1.18) |
|  |  |  |  |  |
| Male | Pre-pregnancy | DASH | Early-childhood overweight & obesity | 1.06 (0.76, 1.48) |
| Female | Pre-pregnancy | DASH | Early-childhood overweight & obesity | 0.94 (0.79, 1.10) |
|  |  |  |  |  |
| Group | Period | Exposure | Outcome | β (95% CI) |
| Male | Pre-pregnancy | DASH | Mid-childhood  fat free mass index | 0.07 (0.01, 0.13)* |
| Female | Pre-pregnancy | DASH | Mid-childhood  fat free mass index | -0.01 (-0.07, 0.06) |

E-DII, energy-adjusted Dietary Inflammatory Index; DASH, Dietary Approaches to Stop Hypertension

**P* <0.05
